# The Genus *Pinnularia* Ehrenberg (Bacillariophyta) from the Transbaikal Area (Russia, Siberia): Description of Seven New Species on the Basis of Morphology and Molecular Data with Discussion of the Phylogenetic Position of *Caloneis*

**DOI:** 10.3390/plants12203552

**Published:** 2023-10-12

**Authors:** Maxim Kulikovskiy, Anton Glushchenko, Elena Kezlya, Irina Kuznetsova, John Patrick Kociolek, Yevhen Maltsev

**Affiliations:** 1 K.A. Timiryazev Institute of Plant Physiology RAS, IPP RAS, 35 Botanicheskaya St., 127276 Moscow, Russia; chelovek91_91@mail.ru (A.G.); melosira@mail.ru (E.K.); pantao@yandex.ru (I.K.); maltsev.ye@yandex.ru (Y.M.); 2 Museum of Natural History, Henderson Building, 15th and Broadway, Boulder, CO 80309, USA; patrick.kociolek@colorado.edu

**Keywords:** Bacillariophyceae, new species, *Pinnularia*, Transbaikal area, systematics, morphology, phylogeny

## Abstract

Seven *Pinnularia* species from the Transbaikal area, Russia, are described as new for science. These are *P. baicalgenkalii*, *P. baicalflexuosa*, *P. microfrauenbergiana*, *P. pergrunowii*, *P. siberiosinistra*, *P. baicalodivergens*, and *P. baicalislandica*. All species are described by original LM and SEM microphotographs and molecular phylogeny. We provide comparisons between the taxa and document variability in the features found in the species. The number of *Pinnularia* species in the Transbaikal area is the largest number of species of the genus anywhere in the world.

## 1. Introduction

As is known, Lake Baikal is the world’s largest freshwater lake, famous for its high biodiversity in many groups of organisms and abundance of endemic species [1,2]. The research of microalgae was started in the 19th century, and the first works already demonstrated the high diversity and peculiarity of diatoms in the bottom sediments [3,4,5,6,7,8,9,10,11]. According to Pomazkina et al. [12], already at the time about 800 diatom taxa had been found, 40% of which were endemic. In a review summarizing the results of long-standing research, Popovskaya et al. [13] indicate that diatoms comprise 49 taxa from 19 genera. Notably, the dominant taxa are exclusively endemic [13].

*Pinnularia* (1843) is one of the most diverse genera of diatoms and currently includes 861 accepted species names, 503 accepted varieties, and 126 accepted formae [14,15]. However, in the publications dedicated to Lake Baikal, information on representatives of this genus is limited. Among the literature sources from the beginning of the 20th century that are available to us, the most comprehensive list of taxa is provided in a review of works by Boris Skvortzov (Skvortzow), featured in vol. 23 of Iconographia Diatomologica [16]. A significant part of Skvortzov’s research was dedicated to the diatoms of Lake Baikal, including deepwater species (from the depth of 30–33 m). He described many new species and varieties. The author of the review Maria Gololobova studied about 60 publications and compiled a list of Skvortzov taxa that comprised 1562 diatom names [17]. From those, 333 taxa are noted for Lake Baikal, including only 21 *Pinnularia* taxa. Meanwhile, *Navicula* (58 taxa), *Cymbella* (35), and *Didymosphaenia* (25) are represented more diversely. Other works of that period mention no more than two to three taxa from this genus. For example, in the list of new and interesting diatoms from Baikal compiled by Vladislav Jasnitsky [10], only two taxa *Pinnularia* are noted, previously described by Skvortzov: *P. hemiptera* (Kützing) Cleve var. *baicalensis* Skvortzov and *P. passargei* Reich var. *baicalensis* Skvortzov. In a work dedicated to diatoms from the periphyton of the northern part of Lake Baikal, Alexander Skabichevsky (Skabichevskij) describes new species, three of which belong to *Pinnularia*: *P. braunii* (Grunow) Cleve var. *scabrosa* Skab., *P. polyonca* (Brebisson) O. Müller var. *scabrosa* Skab., and *P. timofeevii* Skab. It is worth noting that all of them are considered rare species and were found at the depth of 26 m [11]. The results of the processing of samples gathered by Niels Foged in 1975 were published posthumously [1], edited by Hannelore Håkansson. Out of 260 taxa, only 7 belong to *Pinnularia* (five species and two varieties). All were described as oligohalobous (indifferent), pH-circumneutral, and cosmopolitan. The taxa were found in samples from the Angara, which is the only river that drains out of the lake. Many taxa were observed from other genera: *Navicula* (37 taxa), *Gomphonema* (29), *Nitzschia* (20), and *Cymbella* (20). H. Håkansson notes that the samples were in general less diverse than those described earlier “by Skvortzow and Meyer (1928) and particularly by Skvortzow (1937)” [8,9].

Currently, the unique algal flora of Lake Baikal is being intensively studied. Several monographs were published during the last two decades, dedicated to the results of long-term studies of diatoms from plankton [18], the littoral zone [19,20], and benthos [21]. The results of the study of samples collected by Skabitschewsky in July 1965 and samples collected during a Darwin Initiative project in 1997 were presented in volumes 23 and 26 of Iconographia Diatomologica [22,23]. Only in these works, 22 new genera and 554 new species were described. Publications dedicated to descriptions of new taxa from Baikal come out regularly. However, representatives of *Pinnularia* are not mentioned in these works. Brief reports are presented in several works by Pomazkina et al. on microphytobenthos [24,25,26]. In south Baikal, *P. microstauron* (Ehrenberg) Cleve is mentioned among taxa that are dominant in winter, and *P. brevicostata* Cleve is often found [24]. During a study in Olkhon Gate and Maloe More straits, representatives of *Pinnularia* were reported only as single finds. Only four species were found: *P. major* var. *hyalina* (Hust.) Skab. and *P. pectinalis* var. *rostrata* Skvortzow, as well as the endemic *P. braunii* var. *scabrosa* Skab. and *P. timofeevii* Skab. [25]. In a publication on the microphytobenthos of Lake Baikal in areas close to rivers, the diversity of this genus is mentioned; however, there is no information on the number of species or list of taxa [26].

Research of diatoms from Baikal that includes the molecular genetic approach is still scarce. So far, only six new species from the genera *Geissleria*, *Sellaphora*, *Placoneis*, *Cymbopleura*, and *Cymbella* have been described with the use of molecular data [27,28,29,30,31]. For five more previously known species from the genera *Planothidium, Stauroneis*, *Craticula*, and *Stephanodiscus,* there are mentions of genetic sequences of strains from Baikal [32,33,34]. In the studies dedicated to the diversity of protist communities in Lake Baikal using metabarcoding, there are only brief reports on diatoms [35,36,37].

The aim of this publication is the molecular investigation and description of the morphology of seven new *Pinnularia* species from the Transbaikal area.

## 2. Results and Discussion

### 2.1. Morphology and Ultrastructure

The studies performed with light and scanning electron microscopy showed that the isolates belong to the new species *Pinnularia baicalgenkalii*, *P. baicalflexuosa*, *P. microfrauenbergiana*, *P. pergrunowii*, *P. siberiosinistra*, *P. baicalodivergens*, and *P. baicalislandica*.

**Figure 1:**
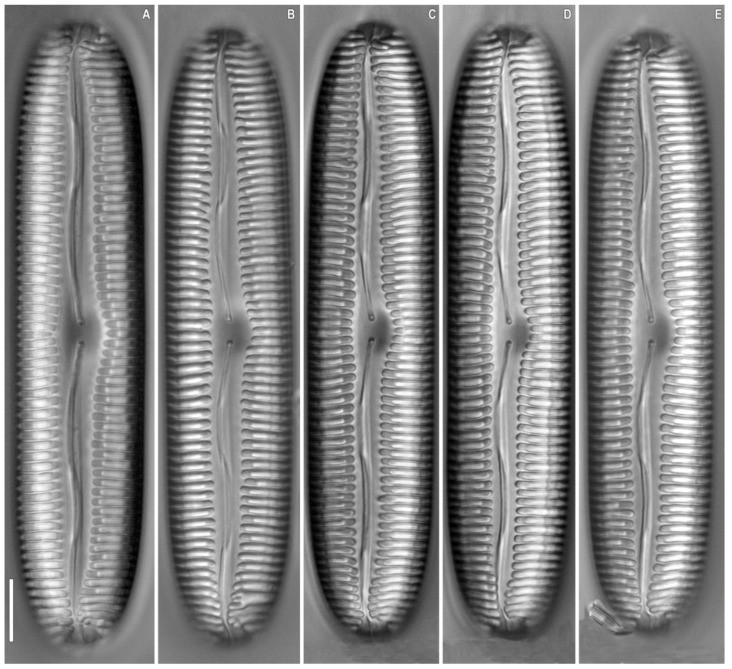
*Pinnularia baicalgenkalii* Kulikovskiy, Glushchenko, Kezlya and Maltsev sp. nov. Strain B194. Oxidized material, Slide No. 19179. (**A**–**E**). Light microscopy, differential interference contrast, size diminution series. (**C**). Holotype. Scale bars = 10 μm.

**Figure 2:**
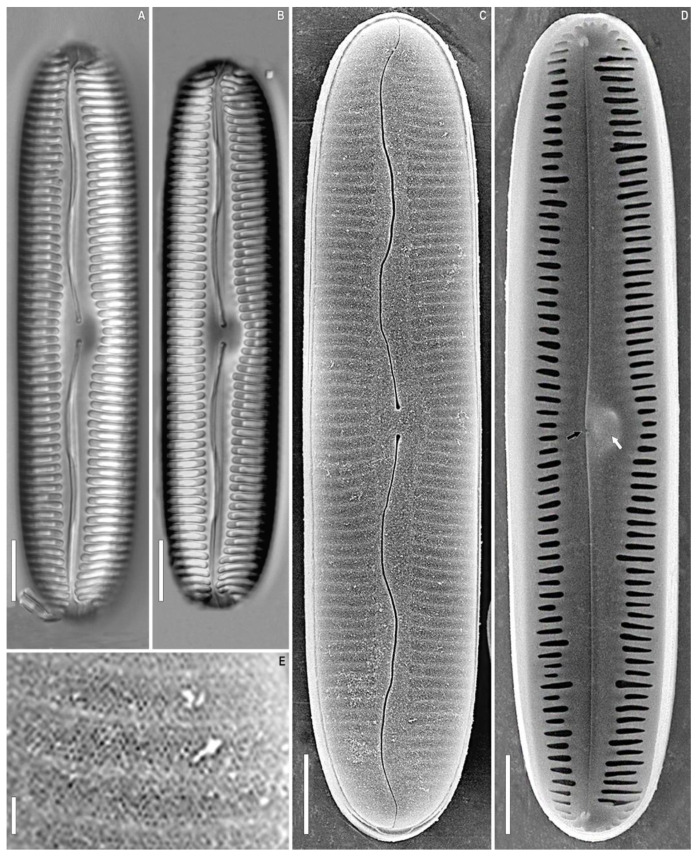
*Pinnularia baicalgenkalii* Kulikovskiy, Glushchenko, Kezlya and Maltsev sp. nov. Strain B194. Oxidized material, Slide no. 19179. (**A**,**B**). Light microscopy, differential interference contrast, size diminution series. (**C**). Scanning electron microscopy, external views. (**D**). Scanning electron microscopy, internal views. (**E**). Scanning electron microscopy, external views, areolae. Scale bars (**A**–**D**) =10 μm; (**E**) =1 μm.

***Pinnularia baicalgenkalii*** Kulikovskiy, Glushchenko, Kezlya and Maltsev sp. nov. (Figure 1 and Figure 2).

**Description**. LM (Figure 1A–D and Figure 2A,B). Valve outline elliptic with parallel sides and broadly rounded ends. Length 92–99 µm, width 19.5–20.0 µm. Axial area linear, narrow, tapering on the ends and widening towards the central area. Central area small, asymmetrically elliptic. Raphe complex, undulate. Striae weakly radiate at the center, becoming parallel to slightly convergent at the ends, 6 in 10 µm.

SEM, external view (Figure 2C). Proximal raphe ends are drop-like and deflected to the same side, but to the opposite direction than the terminal ends. Terminal raphe fissures are externally hooked and unilaterally deflected, reaching the valve mantle at the apex. Striae alveolate, multiseriate (about 7–9 areolae rows).

SEM, internal view (Figure 2D). Central raphe ends are uninterrupted with knot and notch (shown by arrows), raphe branches end in polar simple helictoglossae, deflected to one side. Alveolar openings are covered 2/3 by an axial plate and 1/3 by a mantle plate, leaving an internal opening which is shorter than the entire alveolus. In LM, this covering gives the impression of two longitudinal lines. Striae are composed of areolae with irregularly rounded openings.

**Holotype here designated**: Slide no. 19179, Figure 1C, from oxidized culture strain no. B194, isolated from sample no. 34, deposited in herbarium of MHA, Main Botanical Garden, Russian Academy of Science, Moscow, Russia.

**Isotype**. Slide no. 19179a, collection of Maxim Kulikovskiy at the Herbarium of the Institute of Plant Physiology, Russian Academy of Science, Moscow, Russia.

**Reference strain**. B194, isolated from the sample no. 34, deposited in the collection of Maxim Kulikovskiy at the Herbarium of the Institute of Plant Physiology, Russian Academy of Sciences, Moscow, Russia.

**Type locality.** Russia, Kapustinskaja River, flowing into the Baikal Lake, near the cape Tolstoj, sample no. 34, benthos (52°38.484′ N 107°23.218′ E), collected by M. Kulikovskiy, 17 July 2011.

**Sequence data**. Partial 18S rDNA gene sequence comprising V4 domain sequence (GenBank accession number KM350092) and partial *rbc*L sequence (GenBank accession number KM350002) for the strain B194.

**Etymology**. The species is named for the species locality, Lake Baikal, and the similarity with *Pinnularia genkalii* Krammer & Lange-Bertalot.

**Distribution**. As yet known only from the type locality.

**Comments**. *P. baicalgenkalii* sp. nov. is similar to *P. reichardtii* Krammer, differing from it by wider valves (19.5–20.0 μm in new species vs. 14.7–18.8 μm in *P. reichardtii*) and lower stria density (6 in 10 μm in new species vs. 8–9 in 10 μm in *P. reichardtii*). The valve ends are blunter in our species, while in *P. reichardtii* they are wide and bluntly rounded. *P. baicalgenkalii* sp. nov. is also similar to *P. genkalii* Krammer & Lange-Bertalot. They have the same stria pattern and structure of the axial and central areas, and the morphometric features overlap (75–130 μm length, 17–20 μm width, 6–7 striae in 10 μm in *P. genkalii* vs. 92–99 μm length, 19.5–20.0 μm width, 6 striae in 10 μm in the new species). *P. baicalgenkalii* sp. nov. can be distinguished by the valve shape (elliptic with length-to-width ratio about 4.70–4.95 in *P. baicalgenkalii* sp. nov. vs. linear with length-to-width ratio about 5.4 in *P. genkalii*). Another species that is morphologically similar to *P. baicalgenkalii* sp. nov. is *P. ilkaschoenfilderi* Krammer (Table 1); however, the valves of the latter species are relatively narrow (length-to-width ratio 5.9 vs. 4.70–4.95 in *P. baicalgenkalii* sp. nov.), have differently shaped valve ends (cuneiform rounded vs. broadly rounded in the new species), and central areas (large, roundish vs. small, asymmetrically elliptic respectively).

**Figure 3:**
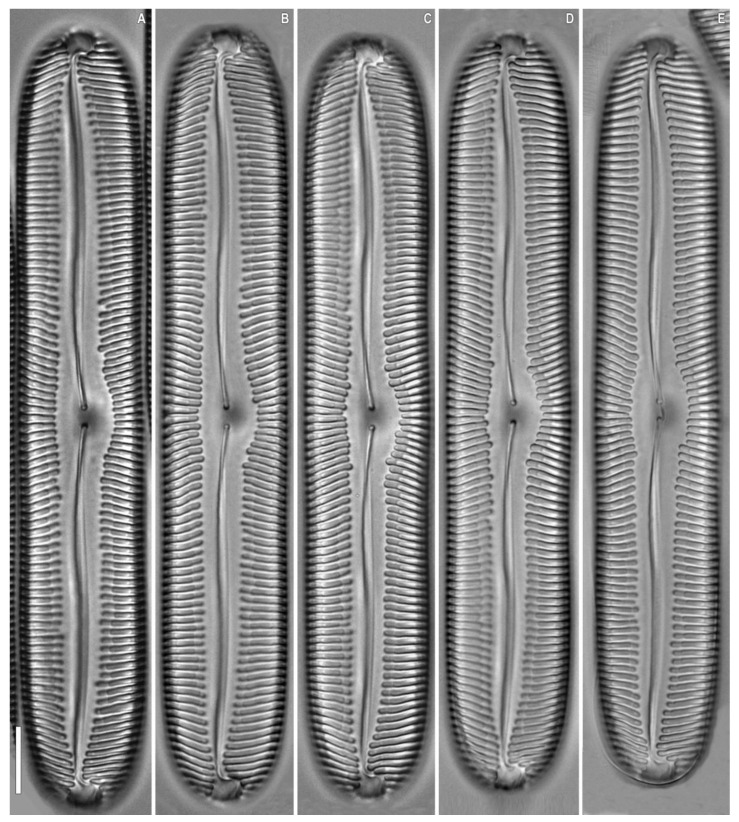
*Pinnularia baicalflexuosa* Kulikovskiy, Glushchenko, Kezlya and Maltsev sp. nov. Strain B054–3. Oxidized material, Slide no. 18959. (**A**–**E**). Light microscopy, differential interference contrast, size diminution series. (**C**). Holotype. Scale bar = 10 μm.

**Figure 4:**
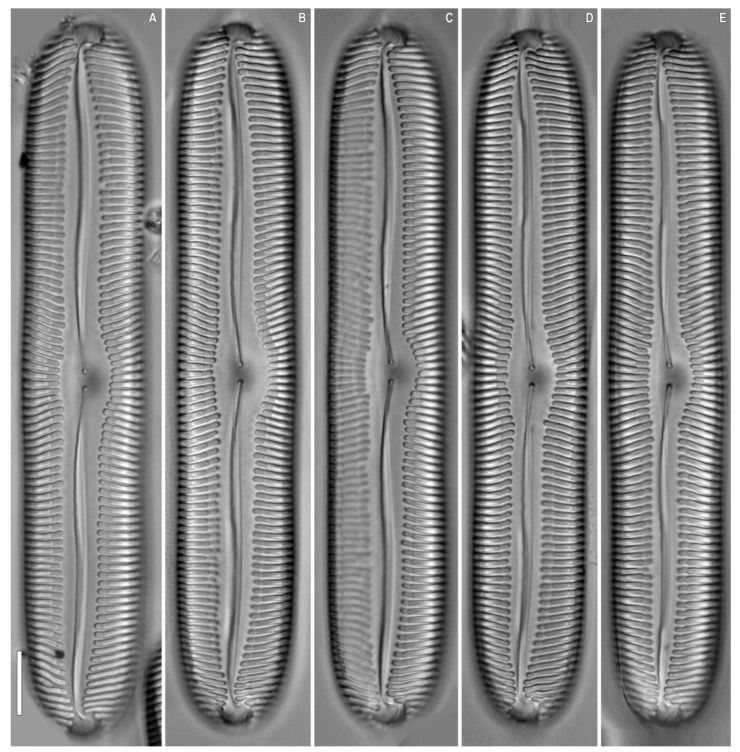
*Pinnularia baicalflexuosa* Kulikovskiy, Glushchenko, Kezlya and Maltsev sp. nov. Strain B054–3. Oxidized material, Slide no. 18959. (**A**–**E**) Light microscopy, differential interference contrast, size diminution series. Scale bar = 10 μm.

**Figure 5:**
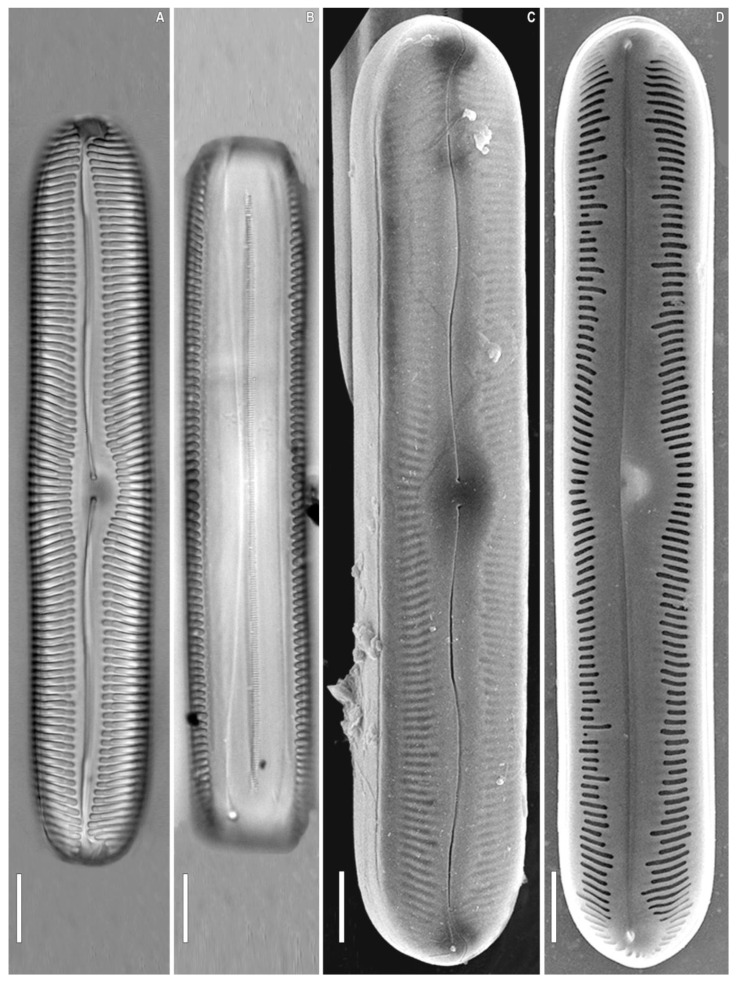
*Pinnularia baicalflexuosa* Kulikovskiy, Glushchenko, Kezlya and Maltsev sp. nov. Strain B054–3. Oxidized material, Slide no. 18959. (**A**,**B**). Light microscopy, differential interference contrast, size diminution series. (**B**). Frustule in girdle view. (**C**). Scanning electron microscopy, external views. (**D**). Scanning electron microscopy, internal views. Scale bars = 10 μm.

***Pinnularia baicalflexuosa*** Kulikovskiy, Glushchenko, Kezlya and Maltsev sp. nov. (Figure 3, Figure 4 and Figure 5).

**Description**. LM (Figure 3A–E, Figure 4A–E and Figure 5A,B). Frustule rectangular in girdle view (Figure 5B). Valve outline linear with parallel sides and broadly rounded ends. Length 109–116 µm, width 17.5–19.0 µm. Axial area linear, narrow, tapering on the ends and widening towards the central area. Central area small, asymmetrically elliptic. Raphe semicomplex, undulate. Striae radiate at the center, becoming convergent at the ends, 7–8 in 10 µm.

SEM, external view (Figure 5C). Proximal raphe ends are drop-like and deflected to the same side but in the opposite direction than the terminal ends. Terminal raphe fissures are externally hooked and unilaterally deflected, reaching the valve mantle at the apex. Striae alveolate, multiseriate (about 5–6 areolae rows).

SEM, internal view (Figure 5D). Central raphe ends are uninterrupted with knot, raphe branches end in polar simple helictoglossae, deflected to one side. Alveolar openings are covered 2/3 by an axial plate and 1/3 by a mantle plate, leaving an internal opening which is shorter than the entire alveolus. In LM, this covering gives the impression of two longitudinal lines.

**Holotype here designated:** Slide no. 18959, Figure 3C, from oxidized culture strain no. B054–3, isolated from sample no. 40, deposited in herbarium of MHA, Main Botanical Garden, Russian Academy of Science, Moscow, Russia.

**Isotype**. Slide no. 18959a, collection of Maxim Kulikovskiy at the Herbarium of the Institute of Plant Physiology, Russian Academy of Science, Moscow, Russia.

**Reference strain**. B054–3, isolated from the sample no. 40, deposited in the collection of Maxim Kulikovskiy at the Herbarium of the Institute of Plant Physiology, Russian Academy of Sciences, Moscow, Russia.

**Type locality**. Russia, Selenga River, near the Brjansk Village, sample no. 40, benthos (52°03.376′ N 106°52.672′ E), collected by M. Kulikovskiy, 19.07.2011.

**Sequence data**. Partial 18S rDNA gene sequence comprising V4 domain sequence (GenBank accession number KM350068) and partial *rbc*L sequence (GenBank accession number KM349984) for the strain B054–3.

**Etymology**. The species is named for the species locality, Lake Baikal, and the similarity with *Pinnularia flexuosa* P.T. Cleve.

**Distribution**. As yet known only from the type locality.

**Comments**. This species is close to *P. flexuosa* P.T. Cleve and differs from it by a lower length (109–116 μm in *P. baicalflexuosa* sp. nov. vs. 173–270 μm in *P. flexuosa*), lower width (17.5–19.0 μm in *P. baicalflexuosa* sp. nov. vs. 32–50 μm in *P. flexuosa*), and a higher stria density (7–8 in 10 μm in *P. baicalflexuosa* sp. nov. vs. 4–5 in 10 μm in *P. flexuosa*) (Table 2). *Pinnularia baicalflexuosa* sp. nov. can be easily confused with *P. neglectiformis* Krammer; the morphometric features overlap in these species [39], (Table 2). They can be delineated by the shape of the valve ends (broadly rounded in *P. baicalflexuosa* sp. nov. vs. cuneiform rounded in *P. neglectiformis*) and the shape of the valves themselves (*P. baicalflexuosa* sp. nov. has valves with parallel sides, whereas the sides of *P. neglectiformis* are slightly convex or triundulate). Another morphologically similar species is *P. torta* (A.Mann) R.M.Patrick, especially the images provided in Liu et al. [40] (p. 149, plate 39, Figures 1–4). However, valves of *P. torta* are larger (length 127.5–189.0 μm, width 20–24 μm vs. 109–116 μm and 17.5–19.0 μm in *P. baicalflexuosa* sp. nov. accordingly).

**Figure 6:**
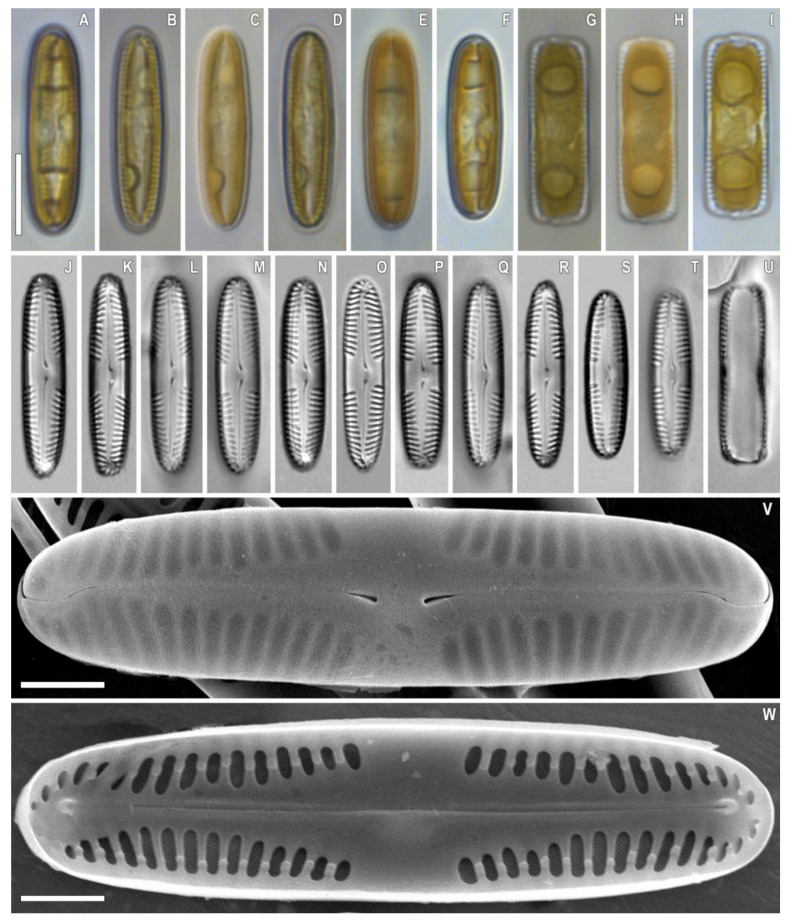
*Pinnularia microfrauenbergiana* Kulikovskiy, Glushchenko, Kezlya and Maltsev sp. nov. Strain B025. Slide no. 18931. (**A**–**U**). Light microscopy, differential interference contrast. (**A**–**I**). Live cells with plastids structure. (**J**–**U**). Oxidized material, size diminution series. (**V**). Scanning electron microscopy, external views. (**W**). Scanning electron microscopy, internal views. (**G**–**I**,**U**). Frustule in girdle view. (**O**). Holotype. Scale bars (**A**–**U**) =10 μm; (**V**,**W**) =2.5 μm.

***Pinnularia microfrauenbergiana*** Kulikovskiy, Glushchenko, Kezlya and Maltsev sp. nov. (Figure 6)

**Description**. LM (Figure 6A–U). Cells solitary, two parallel plastids on either side of the apical axis are present (Figure 6A–I). Frustule rectangular in girdle view (Figure 6G–I,U). Valve outline narrowly elliptical with rounded ends. Length 20–25 µm, width 4.5–5.0 µm. Axial area narrowly lanceolate, widening towards the central area. Central area is represented by a wide transverse fascia. Raphe weakly lateral, filiform, and well noticeable under LM. Striae radiate at the center, becoming convergent at the ends, 14–15 in 10 µm.

SEM, external view (Figure 6V). Proximal raphe ends are drop-like and deflected to the same side but in the opposite direction than the terminal ends. Terminal raphe fissures are externally hooked and unilaterally deflected, reaching the valve mantle at the apex. Striae are alveolate, multiseriate; longitudinal lines are absent.

SEM, internal view (Figure 6W). Central raphe ends are uninterrupted with knot and notch, internal raphe branches end in polar simple helictoglossae, deflected in the same direction. On each of the interstriae there is a single small outgrowth of a rounded or rectangular shape.

**Holotype here designated:** Slide no. 18931, Figure 6O, from oxidized culture strain no. B025, isolated from sample no. 51.1, deposited in herbarium of MHA, Main Botanical Garden, Russian Academy of Science, Moscow, Russia.

**Isotype**. Slide no. 18931a, collection of Maxim Kulikovskiy at the Herbarium of the Institute of Plant Physiology, Russian Academy of Science, Moscow, Russia.

**Reference strain**. B025, isolated from the sample no. 51.1, deposited in the collection of Maxim Kulikovskiy at the Herbarium of the Institute of Plant Physiology, Russian Academy of Sciences, Moscow, Russia.

**Type locality**. Russia, Vydrinnaja River, sample no. 51.1, periphyton (51°29.383’ N 104°50.986′ E), *c*ollected by M. Kulikovskiy, 20 July 2011.

**Sequence data**. Partial 18S rDNA gene sequence comprising V4 domain sequence (GenBank accession number KM350062 and partial *rbc*L sequence (GenBank accession number KM349979) for the strain B025.

**Etymology**. The species is named for the smaller size, and the similarity with *Pinnularia frauenbergiana* Reichardt.

**Distribution**. As yet known only from the type locality.

**Comments**. *Pinnularia microfrauenbergiana* sp. nov. is distinguished from *P. frauenbergiana* Reichardt by the outline, stria density, and axial area, differing in general by stria density (14–15 in 10 μm in *P. microfrauenbergiana* sp. nov. vs. 18–22 in 10 μm in *P. frauenbergiana*). *P. microfrauenbergiana* sp. nov. is also close to *P. bullacostae* Krammer et Lange-Bertalot in Lange-Bertalot & Genkal; however, new species differs by more radiate striae and less blunted valve ends, and its valve sides are not concave as in *P. bullacostae* (Table 3). *Pinnularia microfrauenbergiana* sp. nov. is close to *P*. *pinseelliana* Zidarova, Kopalová & Van de Vijver, described from the Antarctic [42]. However, *P. microfrauenbergiana* sp. nov. does not exhibit protracted valve ends at all, while this feature is present in *P*. *pinseelliana.* The valves of *P*. *pinseelliana* are more widened in the center part relative to the ends than in *P. microfrauenbergiana* sp. nov. Other morphological features are similar in these two species.

An interesting feature of *Pinnularia microfrauenbergiana* sp. nov., *P. bullacostae,* and *P. pinseelliana* is the presence of a morphological structure located on the inner side of the raised interstriae (virgae) that is described by researchers in various ways: “knopfartige höcker” [43], “papillae-like structures” [38], “elevated siliceous outgrowth” [42].

**Table 3:** Comparison of morphological features of *P. microfrauenbergiana* sp. nov. and related species.

	*P. microfrauenbergiana* sp. nov.	* P. frauenbergiana *	* P. bullacostae *	* P. pinseelliana *
**Outline**	narrowly elliptical	linear elliptic-lanceolate with weakly convex sides	linear with parallel up to slightly concave sides	narrowly lanceolate with weakly but still markedly convex, never parallel margins
**Ends**	rounded	evenly tapered and not differentiated	broadly cuneiform rounded	non- to weakly protracted, never capitate nor rostrate, broadly rounded
**Length, μm**	20–25	17–34	33–34	24–30
**Width, μm**	4.5–5	4.3–4.8	5.8–6.7	4.5–5.5
**Striae in 10 μm**	14–15	18–22	15	13–15
**Raphe**	weakly lateral, filiform	filiform	narrow, lateral	lateral, with straight to weakly curved branches
**Axial area**	narrowly lanceolate, widening towards the central area	broadly lanceolate	narrow	narrow near the apices, gradually but distinctly widening towards the central area
**Central area**	represented by a wide transverse fascia	represented by a wide transverse fascia	small fascia	wedge-shaped (rarely rectangular) fascia
**References**	This study	[38,44]	[38,43]	[42]

**Figure 7:**
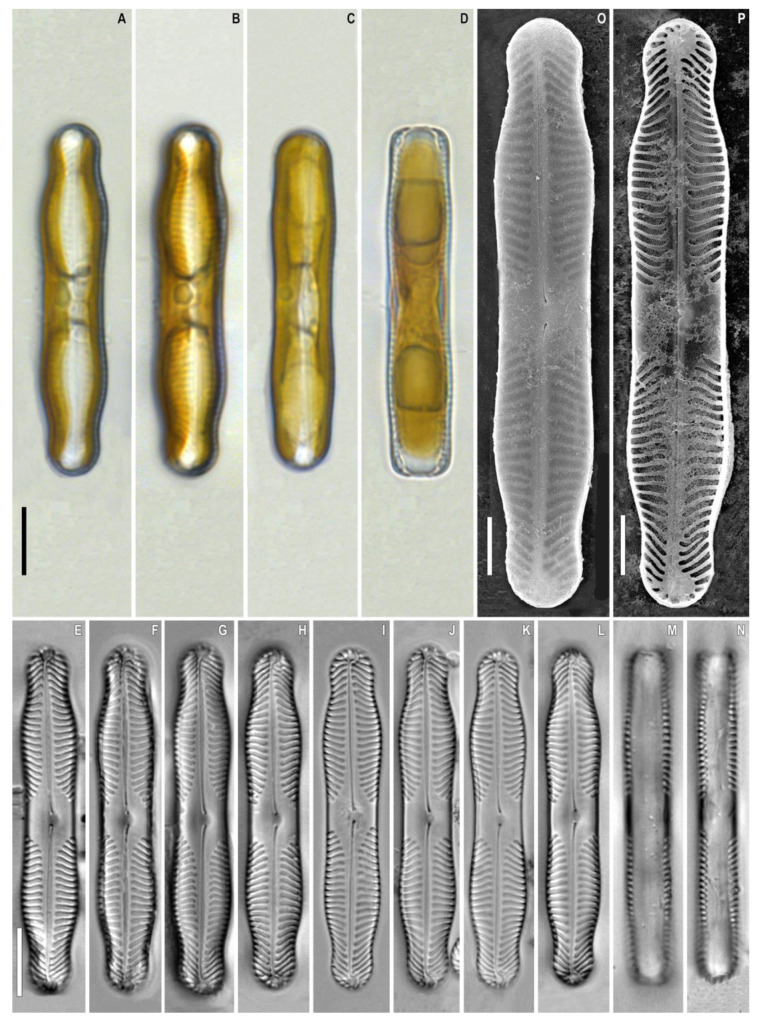
*Pinnularia pergrunowii* Kulikovskiy, Glushchenko, Kezlya and Maltsev sp. nov. Strain B 162–3. Slide no. 18989. (**A**–**N**). Light microscopy, differential interference contrast. (**A**–**D**). Live cells with plastids structure. (**E**–**N**). Oxidized material, size diminution series. (**O**). Scanning electron microscopy, external views. (**P**). Scanning electron microscopy, internal views. (**D**,**M**,**N**). Frustule in girdle view. (**H**). Holotype. Scale bars (**A**–**N**) =10 μm; (**O**,**P**) =5 μm.

***Pinnularia pergrunowii*** Kulikovskiy, Glushchenko, Kezlya and Maltsev sp. nov. (Figure 7).

**Description**. LM (Figure 7A–E). Cells solitary, two parallel plastids on either side of the apical axis are present (Figure 7A–D). Frustule rectangular in girdle view (Figure 7D,M,N) with slightly undulating margins. Valve outline linear with capitate ends and shoulders that are broader than the central part. Length 49.5–51.0 µm, width of central part 7 µm, width of shoulders 8.0–8.5 µm. Axial area linear, narrow and widening towards the central area. Central area is represented by a wide transverse fascia. Raphe straight, filiform, and well noticeable under LM. Striae strongly radiate to radiate at the center, becoming convergent to strongly convergent towards the ends, 10–11 in 10 µm.

SEM, external view (Figure 7F). Proximal raphe ends are drop-like and deflected to the same side but in the opposite direction than the terminal ends. Terminal raphe fissures are externally hooked and unilaterally deflected, reaching the valve mantle at the apex. Striae are alveolate, multiseriate, composed of areolae with irregularly rounded openings.

SEM, internal view (Figure 7G). Central raphe ends are continuous with knot; raphe branches end in polar simple helictoglossae. There is no covering over the alveoli. Longitudinal lines are absent.

**Holotype here designated:** Slide no. 18989, Figure 7H, from oxidized culture strain no. B162–3, isolated from sample no. 28.2, deposited in herbarium of MHA, Main Botanical Garden, Russian Academy of Science, Moscow, Russia.

**Isotype**. Slide no. 18989a, collection of Maxim Kulikovskiy at the Herbarium of the Institute of Plant Physiology, Russian Academy of Science, Moscow, Russia.

**Reference strain**. B162–3, isolated from the sample no. 28.2, deposited in the collection of Maxim Kulikovskiy at the Herbarium of the Institute of Plant Physiology, Russian Academy of Sciences, Moscow, Russia.

**Type locality**. Russia, Bolshaya Suhaya River near the Zarech’e Settlement, sample no. 28.2, benthos (52°33.418′ N 107°08.564′ E), collected by M. Kulikovskiy, 17.07.2011.

**Sequence data**. Partial 18S rDNA gene sequence comprising V4 domain sequence (GenBank accession number KM350084 and partial *rbc*L sequence (GenBank accession number KM349996) for the strain B162–3.

**Etymology**. The species is named for the similarity with *Pinnularia grunowii* Krammer.

**Distribution**. As yet known only from the type locality.

**Figure 8:**
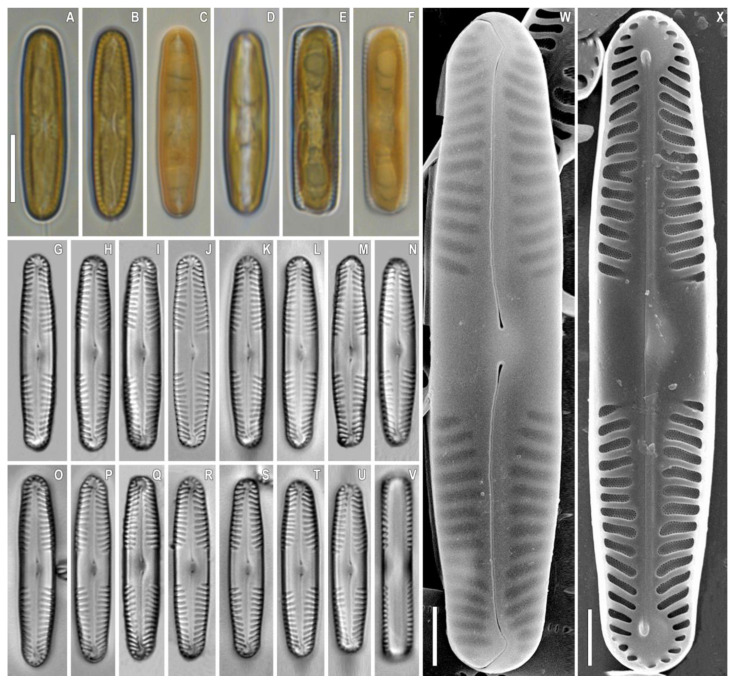
*Pinnularia siberiosinistra* Kulikovskiy, Glushchenko, Kezlya and Maltsev sp. nov. Strain B024–1. Slide no. 18930. (**A**–**V**). Light microscopy, differential interference contrast. (**A**–**F**). Live cells with plastids structure. (**G**–**V**). Oxidized material, size diminution series. (**W**). Scanning electron microscopy, external views. (**X**). Scanning electron microscopy, internal views. (**E**,**F**,**V**). Frustule in girdle view. (**H**). Holotype. Scale bars (**A**–**V**) =10 μm; (**W**,**X**) =2.5 μm.

**Figure 9:**
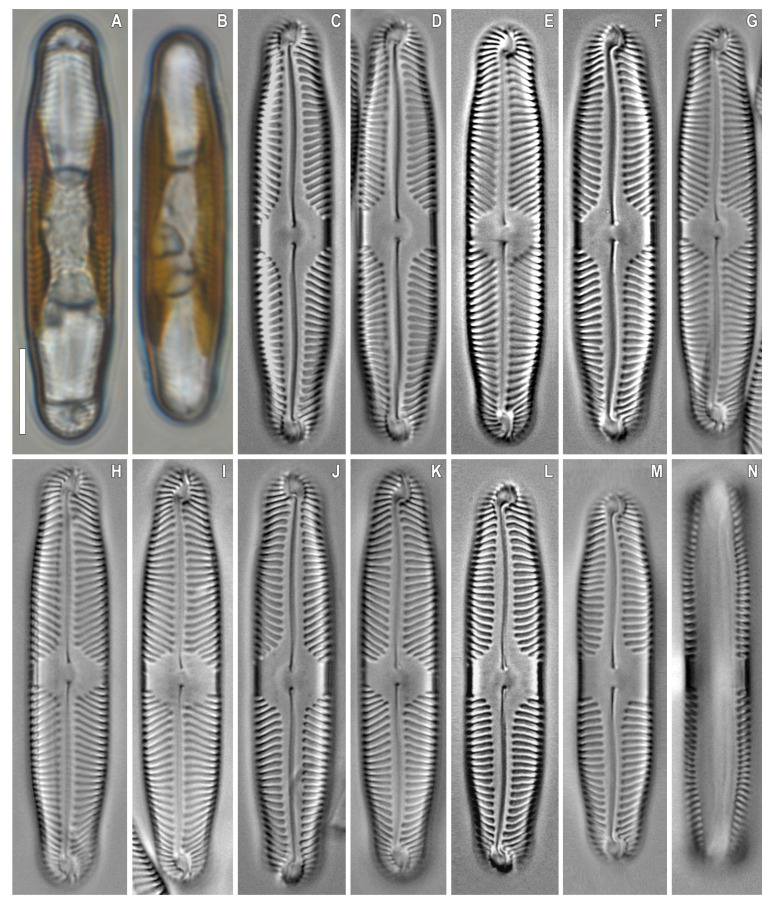
*Pinnularia baicalodivergens* Kulikovskiy, Glushchenko, Kezlya and Maltsev sp. nov. Strain B 112. Slide no. 19170. Light microscopy, differential interference contrast. (**A**,**B**). Live cells with plastids structure. (**C**–**N**). Oxidized material, size diminution series. (**N**). Frustule in girdle view. (**C**). Holotype. Scale bar = 10 μm.

**Figure 10:**
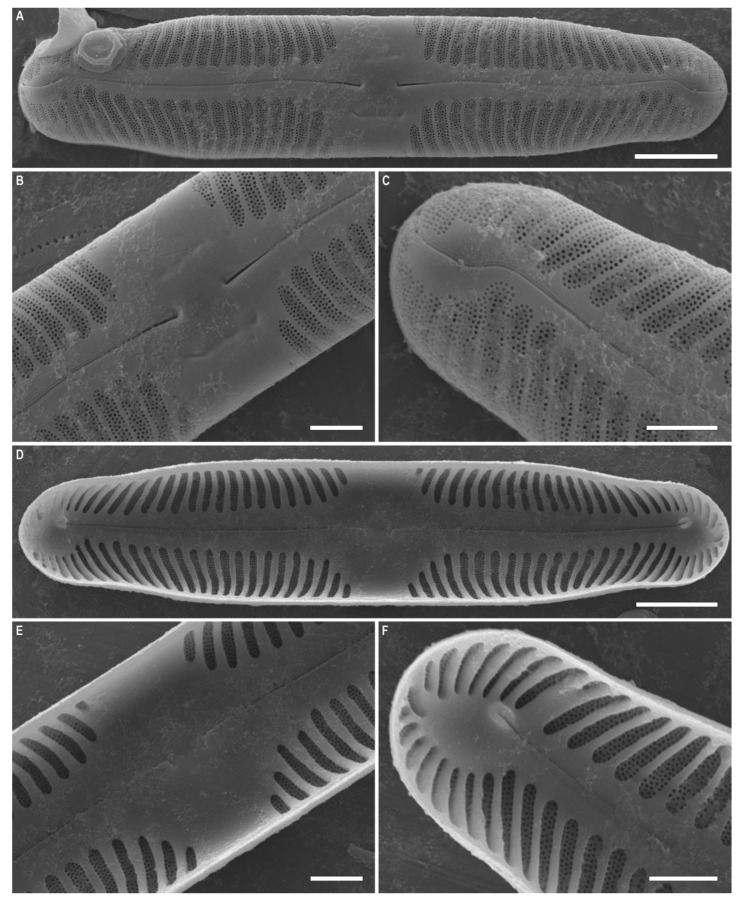
*Pinnularia baicalodivergens* Kulikovskiy, Glushchenko, Kezlya and Maltsev sp. nov. Oxidized material, Strain B112. Scanning electron microscopy. (**A**–**C**). External views. (**D**–**F**). Internal views. (**A**,**D**). The whole valve. (**B**,**E**). Central area. (**C**,**F**). Valve end. Scale bars (**A**,**D**) =5 μm; (**B**,**C**,**E**,**F**) =2 μm.

**Figure 11:**
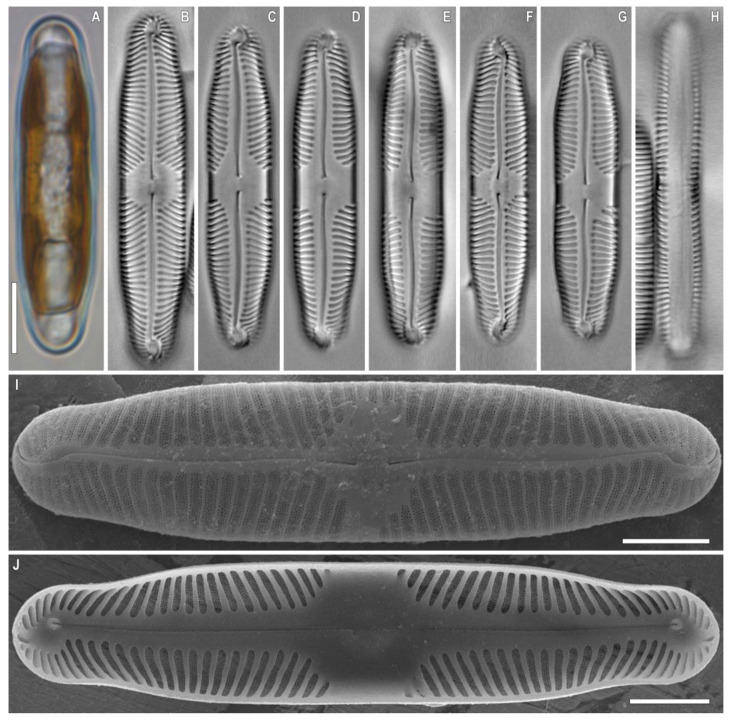
*Pinnularia baicalodivergens* Kulikovskiy, Glushchenko, Kezlya and Maltsev sp. nov. Oxidized material, Strain B097. (**A**–**H**). Light microscopy, differential interference contrast. (**A**). Live cell with plastids structure. (**B**–**H**). Slide no. 19248, oxidized material, size diminution series. (**I**). Scanning electron microscopy, external views. (**J**). Scanning electron microscopy, internal views. (**H**). Frustule in girdle view. Scale bars (**A**–**H**) =10 μm.; (**I**,**J**) =5 μm.

**Figure 12:**
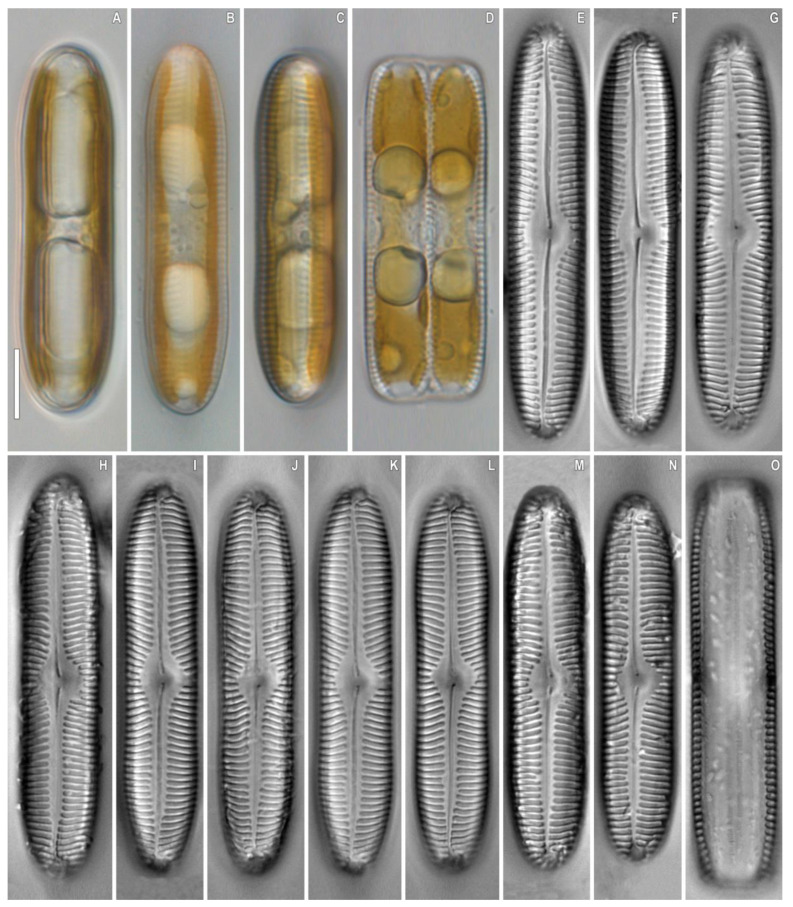
*Pinnularia baicalislandica* Kulikovskiy, Glushchenko, Kezlya and Maltsev sp. nov. Strain B238. Slide no. 18997. Light microscopy, differential interference contrast, (**A**–**D**). Live cells with plastids structure. (**E**–**O**). Oxidized material, size diminution series. (**D**,**O**). Frustule in girdle view. (**E**). Holotype. Scale bars = 10 μm.

**Figure 13:**
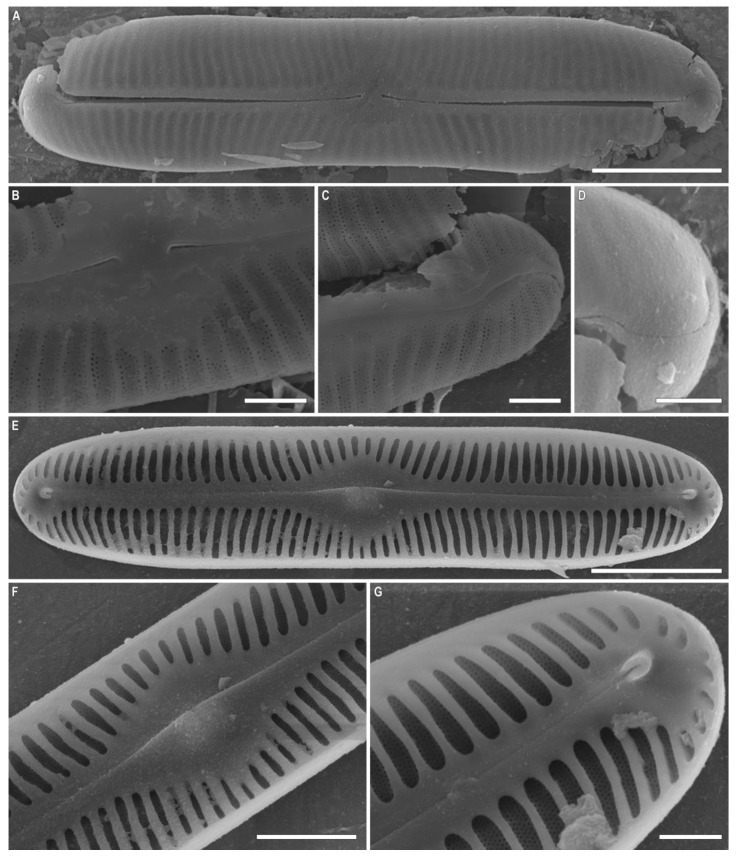
*Pinnularia baicalislandica* Kulikovskiy, Glushchenko, Kezlya and Maltsev sp. nov. Strain B238, oxidized material. Scanning electron microscopy. (**A**–**D**). External views. (**E**–**G**). Internal views. Scale bars (**A**,**E**) =10 μm; (**F**) =5 μm; (**B**–**D**,**G**) =2 μm.

**Figure 14:**
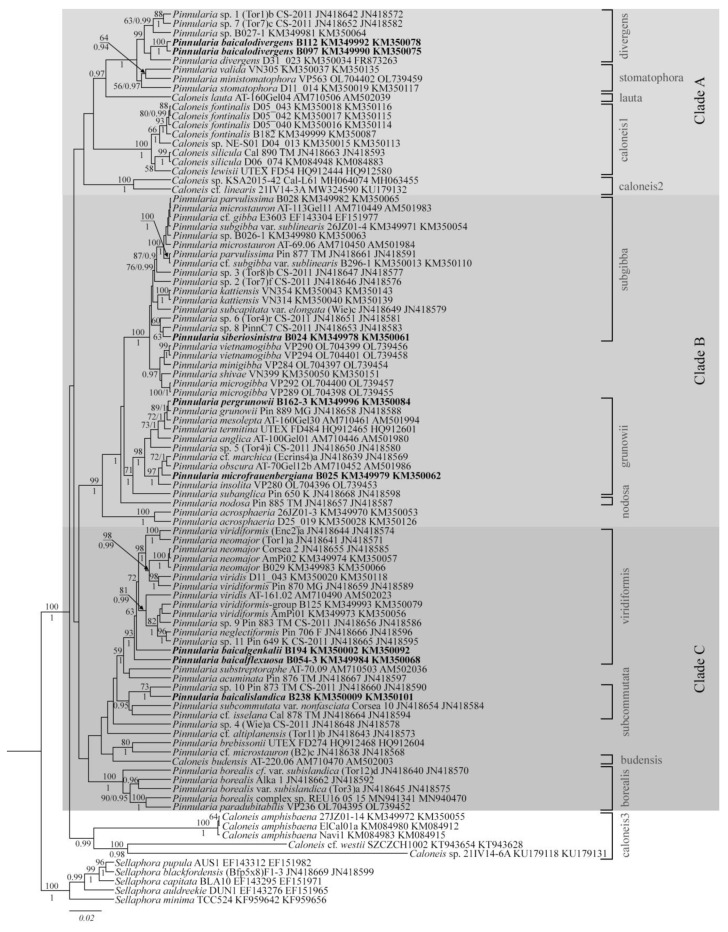
Phylogenetic position of the new *Pinnularia* species (indicated in bold) based on Bayesian inference for the partial *rbc*L and 18S rRNA genes. The total length of the alignment is 1797 characters. Bootstrap supports (BS) from ML (constructed by RA × ML) and posterior probabilities (PP) from BI (constructed by Beast) are presented on the nodes in order. Only BS and PP above 50 and 0.9 are shown. Strain numbers (if available) and GenBank numbers are indicated for all sequences.

**Comments**. *Pinnularia pergrunowii* sp. nov. is close to *P. grunowii* Krammer (Table 4); however, *P. grunowii* has distinctly triundulate sides of the valve, while new species has a small constriction at the center of the valve. The valve ends are less constricted in our species than in *P. grunowii.* The stria density in *P. pergrunowii* is generally lower than in *P. grunowii* (10–11 in 10 μm in *P. pergrunowii* vs. 11–14 in 10 μm in *P. grunowii*). Among similar species that are the same size, with capitate ends and a fascia, we should note *P. rhombofasciata* Krammer & Metzeltin and *P. dicephala* (Ehrenberg) W. Smith (Table 4). *P. pergrunowii* can be easily differentiated from these species by the concave sides. Biundulate valves of *P. ferrophila* Krammer are similarly shaped but can be differentiated by the valve width (8.0–8.5 μm in *P. pergrunowii* sp. nov. vs. 8.8–10 μm in *P. ferrophila*). Also, we should note the similarity of *P. pergrunowii* sp. nov. with illustrations of *P. latarea* Krammer provided by Siver et al. [45] (p. 581, plate 168, Figures 1–14). These species are similar in size and valve shape (linear with concave sides and capitate ends) (Table 4).

A clear difference is in the structure of the axial and central area. In *P. latarea,* they form together a wide, lanceolate space with a very broad fascia, whereas in *P. pergrunowii* sp. nov. the axial area is distinctly separated, narrow, linear. The valve sides in *P. pergrunowii* are more concave than in *P. latarea*. We also studied vouchers of strains (images and metadate on the culture collection website or in publications associated with the nucleotide sequence) close in phylogenetic position: *P. anglica* AT100Gel01, *P. mesolepta* AT_160Gel30, *P. grunowii* Pin 889 MG, *P. termitina* UTEX FD484. All of them are clearly different from the new species (Table 4).

***Pinnularia siberiosinistra*** Kulikovskiy, Glushchenko, Kezlya and Maltsev sp. nov. (Figure 8).

**Description**. LM (Figure 8A–V). Cells solitary, two parallel plastids on either side of the apical axis are present (Figure 8A–F). Frustule rectangular in girdle view (Figure 8E,F,V). Valve outline narrowly elliptical with subcapitate ends. Length 25–29 µm, width 5 µm. Axial area narrowly lanceolate and widening towards the central area. Central area is represented by a wide transverse fascia. Raphe straight, filiform, and weakly noticeable under LM. Striae strongly radiate to radiate at the center, becoming convergent to strongly convergent towards the ends, 12–14 in 10 µm.

SEM, external view (Figure 8W). Proximal raphe ends are drop-like and deflected to the same side but in the opposite direction than the terminal ends. Terminal raphe fissures are externally hooked and unilaterally deflected, reaching the valve mantle at the apex.

SEM, internal view (Figure 8X). Central raphe ends are continuous with knot; internal raphe branches end in polar simple helictoglossae. There is no internal covering over the alveoli. Longitudinal lines are absent. Striae alveolate, multiseriate (about 5–6 areolae rows), composed of areolae with irregularly rounded openings.

**Holotype here designated:** Slide no. 18930, Figure 8H, from oxidized culture strain no. B024–1, isolated from sample no. 51.1 deposited in herbarium of MHA, Main Botanical Garden, Russian Academy of Science, Moscow, Russia.

**Isotype**. Slide no. 18930a, Collection of Maxim Kulikovskiy at the Herbarium of the Institute of Plant Physiology, Russian Academy of Science, Moscow, Russia.

**Reference strain**. B024–1, isolated from the sample no. 51.1, deposited in the collection of Maxim Kulikovskiy at the Herbarium of the Institute of Plant Physiology, Russian Academy of Sciences, Moscow, Russia.

**Type locality**. Russia, Vydrinnaja River, sample no. 51.1, periphyton (51°29.383′ N 104°50.986′ E), collected by M. Kulikovskiy, 20.07.2011.

**Sequence data**. Partial 18S rDNA gene sequence comprising V4 domain sequence (GenBank accession number KM350061) and partial *rbc*L sequence (GenBank accession number KM349978) for the strain B024–1.

**Etymology**. The species is named for the species locality—the extensive geographical region Siberia—and the similarity with *Pinnularia sinistra* Krasske.

**Distribution**. As yet known only from the type locality.

**Comments**. *Pinnularia siberiosinistra* sp. nov. is morphologically similar to *P. sinistra* Krammer (Table 5). It differs from the type population of the Krammer species by a stronger tapering of the valve ends and a more widened axial area than in *P. sinistra* (Table 5). New species could also be compared with the material illustrated by Souffreau et al. [39] (p. 867, Figure 1o) marked as “*P*. sp. (Tor4)r”. *P. siberiosinistra* sp. nov. differs from *P.* sp. (Tor4)r by a narrow elliptical valve shape, while *P.* sp. (Tor4)r has linear valves with slightly undulate ends. Other features are quite similar in these two species (Table 5).

***Pinnularia baicalodivergens*** Kulikovskiy, Glushchenko, Kezlya and Maltsev sp. nov. (Figure 9 and Figure 10).

**Description**. LM (Figure 9A–N). Cells solitary, two parallel plastids on either side of the apical axis are present (Figure 9A,B and Figure 11A). Frustules rectangular in girdle view (Figure 9N and Figure 11H). Valve outline linear with parallel sides and subcapitate rounded ends. Length 46–50 µm, width 8–9 µm. Axial area linear, narrow, tapering at the ends and widening towards the central area. Central area is represented by an asymmetric transverse fascia. On the central area are often present irregular structures as diverse flecks, chaotic or united in a line. Raphe straight, filiform, and well noticeable under LM. Striae strongly radiate to radiate at the center, becoming convergent to strongly convergent at the ends; longitudinal lines are absent, 11–12 in 10 µm.

SEM, external views (Figure 10A–C). Proximal raphe ends are drop-like and deflected to the same side but in the opposite direction than the terminal ends. Terminal raphe fissures are externally hooked and unilaterally deflected, reaching the valve mantle at the apex. On the central area on either side of the central nodule grooves of different sizes and shapes are located. Striae alveolate, multiseriate (about 4–5 areolae rows), composed of areolae with irregularly rounded openings. 

SEM, internal views (Figure 10D–F). Central raphe ends are continuous with knot; internal raphe branches end in polar simple helictoglossae, which are slightly deviated relative to the apical axis of the valve. There is no internal covering over the alveoli. Longitudinal lines are absent.

**Holotype here designated**: Slide no. 19170, Figure 9C, from oxidized culture strain no. B112 isolated from sample no. 4.5, (in collection of Maxim Kulikovskiy, Institute of Plant Physiology, Russian Academy of Sciences, Russia.

**Isotype**. Slide no. 19170a, Collection of Maxim Kulikovskiy at the Herbarium of the Institute of Plant Physiology, Russian Academy of Science, Moscow, Russia.

**Reference strain**. B112, isolated from sample no. 4.5, deposited in the collection of Maxim Kulikovskiy at the Herbarium of the Institute of Plant Physiology, Russian Academy of Sciences, Moscow, Russia.

**Type locality**. Russia, unnamed Bay in 8 km from Jenhaluk, sample no. 4.5, benthos (52°27.042′ N 106°53.215′ E), leg. M. Kulikovskiy, 14.07.2011.

**Sequence data**. Partial 18S rDNA gene sequence comprising V4 domain sequence (GenBank accession number KM350075) and partial *rbc*L sequence (GenBank accession number KM349990) for the strain B112.

**Etymology**. The species is named for the species locality, Lake Baikal, and the similarity with *Pinnularia divergens* W. Smith.

**Distribution**. As yet known only from the type locality.

**Representative specimen**. Strain B097, slide no. B097 (19248) (Figure 11), isolated from sample no. 4.5, collected by M. Kulikovskiy, 14 July 2011. Sequence data: partial 18S rDNA gene sequence comprising V4 domain sequence (GenBank accession number KM350075) and partial *rbc*L sequence (GenBank accession number KM349990).

**Comments**. *Pinnularia baicalodivergens* sp. nov. is distinguished from *P. divergens* W.Smith by the valve outline and dimensions. The most similarity in size and shape is with *P. divergens* var. *media* Krammer (Table 6). However, the valves of *P. baicalodivergens* sp. nov. are slightly narrower (8–9.4 µm in *P. baicalodivergens* sp. nov. vs. 10–13 μm in *P. divergens* var. *media*). Our species is also close to *P. microstauron* var. *microstauron* (Ehrenberg) P.T. Cleve, differing from it mostly by a lower valve width (8–9.4 μm in *P. baicalodivergens* sp. nov. vs. 10–12.4 μm in *P. microstauron* var. *microstauron*). Another morphometrically similar species is *P. submicrostauron* Liu, Kociolek & Q.X. Wang (Table 6). It can be delineated from the new species by the shape of the valve ends: subcapitate in *P. baicalodivergens* sp. nov. vs. rostrate in *P. submicrostauron*.

***Pinnularia baicalislandica*** Kulikovskiy, Glushchenko, Kezlya and Maltsev sp. nov. (Figure 12 and Figure 13).

**Description**. LM (Figure 12A–O). Cells solitary, two parallel plastids on either side of the apical axis are present (Figure 12A–D). Frustule rectangular in girdle view (Figure 12D,O). Valve outline linear with parallel sides and cuneiform rounded ends. Length 54–60 µm, width 10–11 µm. Axial area linear, narrow, tapering on the ends and widening towards the central area. Central area asymmetric, rhomboid. Raphe straight, filiform, and well noticeable under LM. Striae strongly radiate to radiate at the center, becoming parallel to convergent at the ends, 10 in 10 µm.

SEM, external views (Figure 13A–D). Proximal raphe ends weakly extended and deflected to the same side but in the opposite direction than the terminal ends. Terminal raphe fissures are externally hooked and unilaterally deflected, reaching the valve mantle at the apex. Striae alveolate, multiseriate (about 4–5 areolae rows), composed of areolae with irregularly rounded openings.

SEM, internal views (Figure 13E–G). Central raphe ends are continuous with knot; internal raphe branches end in polar simple helictoglossae. There is no internal covering over the alveoli. Longitudinal lines are absent.

**Holotype here designated**: Slide no. 18997, Figure 12E, from oxidized culture strain B238, isolated from sample no. 11.2, deposited in herbarium of MHA, Main Botanical Garden, Russian Academy of Science, Moscow, Russia.

**Isotype**. Slide no. 18997a, Collection of Maxim Kulikovskiy at the Herbarium of the Institute of Plant Physiology, Russian Academy of Science, Moscow, Russia.

**Reference strain**. B238, isolated from sample no. 11.2, deposited in the collection of Maxim Kulikovskiy at the Herbarium of the Institute of Plant Physiology, Russian Academy of Sciences, Moscow, Russia.

**Type locality**. Russia, Zagza River, sample no. 11.2, periphyton (52°31.656’ N 107°05.114′ E), collected by M. Kulikovskiy, 15 July 2011.

**Sequence data**. Partial 18S rDNA gene sequence comprising V4 domain sequence (GenBank accession number KM350101) and partial *rbc*L sequence (GenBank accession number KM350009) for the strain B024–1.

**Etymology**. The species is named for the species locality, Lake Baikal, and the similarity with *Pinnularia islandica* Østrup.

**Distribution**. As yet known only from the type locality.

**Comments**. This species is distinguished from *P. islandica* Østrup by the valve outline and the shape of axial and central areas (Table 7). New species has a lower valve width (10–11 μm vs. 12–14 μm in *P. islandica*). The axial area in *P. baicalislandica* sp. nov. is linear, distinctly separated from the central area, while in the valves of *P. islandica* this separation is not clear, and the axial area is wedge-shaped. New species is distinguished from *P. subcommutata* Krammer and *P. subcommutata* var. *nonfasciata* Krammer by the valve outline (in *P. subcommutata* the sides are slightly convex whereas in *P. baicalislandica* sp. nov. they are parallel). *P. rupestris* Hantzsch differs from *P. baicalislandica* sp. nov. by stria density (12–13 in 10 µm in *P. rupestris* vs. 10 in 10 µm in the new species). *P. perspicua* Krammer can be differentiated by the presence of crescent-like markings on the central area; in *P. baicalislandica* sp. nov. such markings are not found. It may be complicated to differentiate the new species from *P. levkovii* Metzeltin, Lange-Bertalot & Soninkhishig (Table 7); the structure of the axial area should be considered (it is expanded towards the central area in *P. levkovii*, while in *P. baicalislandica* sp. nov. it is linear, narrow). The central area is distinctly separated, asymmetric, rhomboid in *P. baicalislandica* sp. nov., and in *P. levkovii* it is variably shaped and is not clearly differentiated. The stria density is lower in *P. levkovii* than in *P. baicalislandica* sp. nov. (8–10 in 10 µm in *P. levkovii* vs. 10 in 10 µm in *P. baicalislandica* sp. nov.).

### 2.2. Phylogenetic Analysis

Our phylogenetic analysis was carried out using the genetic markers *rbc*L and 18S rDNA and included 76 strains of *Pinnularia* and 17 strains of *Caloneis*. It is these genes that are most often used in work with this group [39,52,53]. In this case, the largest number of nucleotide sequences is available for the *rbc*L gene, somewhat less for 18S rRNA. Significantly fewer sequences are available for 28S rRNA and *cox1* genes. Therefore, we have chosen a strategy for using 18S rRNA and *rbc*L genes to minimize the loss of *Pinnularia* strains and species for which other genes are not available. Previous studies already showed that *Pinnularia* and *Caloneis* form a monophyletic group and there are three well supported clades within this group, designated as A, B, C by Souffreau et al. [39,52,53]. Our phylogeny confirms these findings and new strains are added to the clades and subclades. Strains of *Caloneis amphisbaena, C*. cf. *westii* SZCZCH1002 and *C.* sp. 21IV14_6A formed an additional clade. All new species described in this study occupy separate positions in the corresponding subclades (Figure 14).

Clade A includes subclades “divergens”, “stomatophora”, and three more subclades are formed by representatives of *Caloneis* (Figure 14).

**Figure 15:**
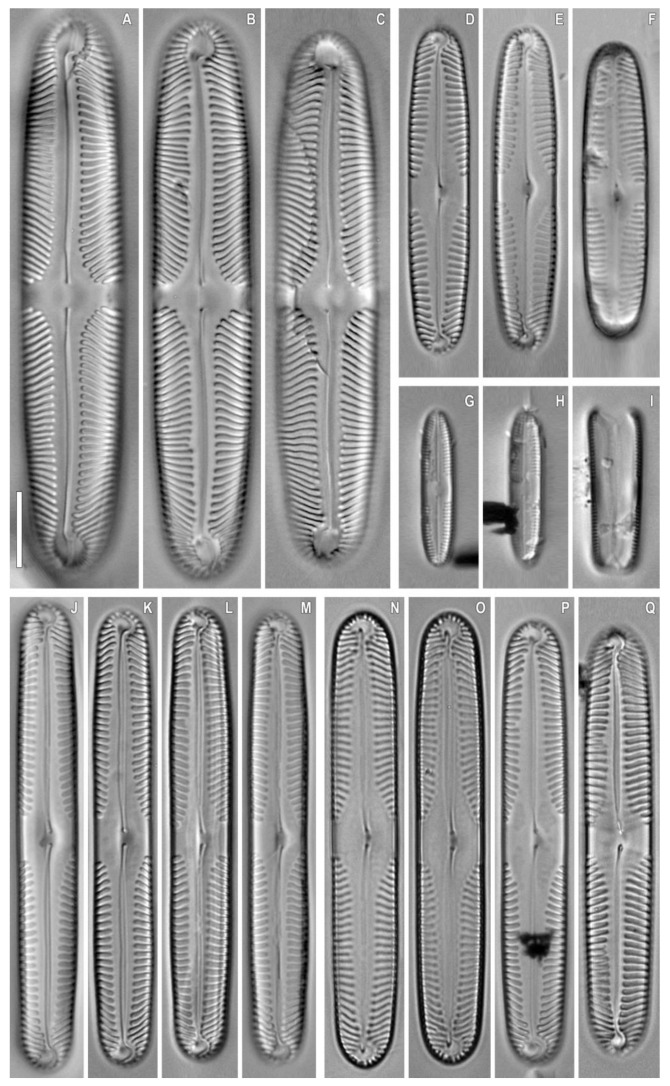
Light micrographs of oxidized material of some strains of *Pinnularia* and *Caloneis* used in our study. (**A**–**C**). *P.* sp., strain B027. (**D**–**F**). *P. parvulissima*, strain B028. (**G**–**I**). *C. fontinalis*, strain B182. (**J**–**M**). *P.* sp., strain B026. (**N**–**Q**). *P. subgibba* var. *sublinearis*, strain B296–1. Scale bar = 10 μm.

Subclade “divergens” includes species that are morphologically close to *P. divergens*, with linear or linear-elliptic valves, capitate, subcapitate or rostrate valve ends, a fascia on the central area, and internally open alveoli. However, a specific feature of the *P. divergens* type is the presence of rounded thickenings at the margin of the fascia. These structures can only be supposedly confirmed for *P.* sp. B027–1 (this study, Figure 15A–C). On the LM image of the *P.* sp. 7 Tor1b voucher thickenings at the margin of the fascia cannot be seen; the LM and SEM images of *P.* sp. 1 Tor7c also do not show any structures on the central area [39] (p. 867, Figure 1a,b,e). On the contrary, in the new species *P. baicalodivergens* we have found crescent-shaped hollow markings on the central area (Figure 10A,B), which brings this species close to representatives of the “stomatophora” subclade.

The “stomatophora” subclade species are united by the presence of well discernible crescent-shaped or irregular hollow markings on the external surface of the central area (*P. stomatophora*, *P. ministomatophora*, *P. valida*).

Subclade “caloneis1” includes representatives of the eponymous genus *C. fontinalis*, *C. silicula*, and *C. lewisii*. The common morphological feature of these species is areolae that are almost fully covered. *C. lauta* AT 160Gel04 form separate line, *C.* sp. KSA2015 with *C.* cf. *linearis* 21IV14 3A form subclade “caloneis 2”.

Clade B includes subclades “subgibba”, “grunowii”, *P. nodosa,* and *P. acrosphaeria* form separate lines.

Most taxa belong to the “subgibba” subclade. A characteristic feature of this subclade for almost all of the taxa is the presence of ghost striae on the inner surface of the central area and fascia.

Vouchers are available and confirm the presence of ghost striae in *P. parvulissima* B028 (this study, Figure 15D–F), *Pinnularia subgibba* var. *sublinearis* B296–1 (this study, Figure 15N–Q), P. *parvulissima* Pin887, *P.* sp. (Tor7) f [39] (p. 867, Figure 1m), *P. vietnamogibba*, *P. minigibba*, and P. *microgibba* [53] (Figure 3, Figure 4, Figure 5, Figure 6, Figure 7 and Figure 8). However, in the new species *P. siberiosinistra,* ghost striae are not found (this study, Figure 8W,X), on the LM image of *P.* sp. 6 Tor4r voucher ghost striae cannot be seen [39] (p. 867), and no image of *P.* sp. 8 PinnC7 is provided in Souffreau et al. [39]. These taxa are grouped in one lineage with maximum statistical support (BS 100, PP 1), and thus its position may change with further addition of data.

Taxa from the “grunowii” subclade do not have any differentiating features in their valve structure, they do not have any markings on the inner or outer surface of the central area. A common feature is an H-shaped chloroplast [39]. Two new species described in the current study are included in this subclade. *P. pergrunowii* occupies a stable position next to *P. grunowii* (Figure 14), and morphologically these species differ clearly (Table 4). 

A small-celled species with an elliptic outline *P. microfrauenbergiana* supplements a high-supported lineage (BS 97, PP 1) of other small-celled species *P. obscura* AT 70Gel12b, *P.* cf. *marchica* Enrins4a., and *P. insolita* VP280.

*P. nodosa* and *P. acrosphaeria* form separate subclades. Subclade “nodosa” now includes only *P. nodosa* Pin855 TM with characteristic markings on the valve sufrace—heavily structured on the outer surface and smooth on the inside [38].

Subclade “acrosphaeria” includes only two strains of *P. acrosphaeria* and is defined by a mottled, distinct structured area on the outer surfaces and slightly structured on the inner surfaces.

In clade C three stable subclades are distinguished: “viridiformis” (BS 93, PP 1) “subcommutata” (BS < 50, PP 0.95), “borealis” (BS 100, PP 1). Several taxa form separate lineage and take up intermediate positions with low support (Figure 14).

Species from subclade “viridiformis” have a complex or semicomplex raphe and round central raphe endings (*P. viridis*, *P. viridiformis*, *P. neomaior*, *P. neglectiphormis,* and the new species *P. baicalgenkalii*, *P. baicaloflexuosa*), whereas species from the “subcommutata” subclade (*P.* cf. *isselana* Cal878TM, *P. subcommutata* var. *nonfasciata* Corsea10, *P.* sp. 10 Pin873TM and P. *baicalislandica* sp. nov.) are characterized by a filiform or lateral raphe with linear central raphe endings. A border lineage between these subclades is currently represented by *P. substreptoraphe* AT70.09 (complex raphe) and *P. acuminata* (lateral raphe, [39] p. 867, Figure 1s), but the support of this lineage is low.

Relatively small species *P. brebissonii* UTEX FD274, *P.* cf. *microstauron* B2c, and *Caloneis budensis* AT220.06 are joined into one group with low support. They differ from the representatives of the “viridiformis” and “subcommutata” subclades by the presence of a fascia. Separate lines between “viridiformis” and “subcommutata” are formed by *P.* sp. 4 (Wie)a and *P. altiplanensis* Tor11b. However, the node supports are quite low. Morphologically, *P.* sp. 4 (Wie)a is closer to the “subcommutata” subclade; it has linear-elliptical valves of medium size (45.8–47.7 µm length and 9.5–9.9 µm width) with a lateral raphe and no fascia [39] (p. 867, Figure 1u), whereas *P. altiplanensis* Tor11b is a small-celled species with a fascia (length 17.4–18.4 µm, width 4.2–4.8 µm, [39] (p. 867, Figure 1x) and thus is closer to the “microstauron” subclade.

Subclade “borealis” is formed by taxa with a characteristic morphotype similar to representatives of the *P. borealis* species complex. Currently the subclade includes taxa with relatively small linear or linear-elliptic valves, widely spaced coarse striae, and a lateral or fusiform raphe.

A new clade “caloneis 3”has been defined with high support, formed by five strains of *Caloneis*. Three strains are represented by the type species of the genus *C. amphisbaena*, they form a subclade with maximum support, and separate lines are formed by *C.* cf. *westii* SZCZCH1002 and *C.* sp. 21IV14–6A.

#### 2.2.1. Phylogeny of the Genera *Pinnularia* and *Caloneis*

The issue of separating *Pinnularia* and *Caloneis* was repeatedly raised by algologists-taxonomists [54,55,56]. *Pinnularia* was described in 1843 by Ehrenberg with the type species *P. viridis* [57]. *Caloneis* was described later by Cleve in 1894 on the basis of longitudinal lines on the valve surface. Even then the unclear differentiation of small-celled forms and a close connection with “some marine, panduriform Pinnulariae” were noted [58]. Nevertheless, to this day, *Caloneis* remains a valid genus and diatomists identify its representatives in flora and describe new species [22,59,60,61]. Traditionally, the genera are delineated by stria density, species with finer and denser spaced striae being attributed to *Caloneis*. According to the opinion of Mann [56] (p. 33), “…there is no adequate basis for the traditional *Pinnularia*-*Caloneis* distinction…”, and more than 100 years “…people learn to recognize not the genus itself, but individual species and species complexes, which they then learn to associate with a particular genus name”. In his discussion of the closeness of *Pinnularia* and *Caloneis* and the great diversity of both genera, Mann from the first page quotes E. Cox, F. Round, and K. Krammer, who speak of the heterogeneity of these genera and of suggestions to divide them into many smaller units.

It would seem that the application of molecular methods would give a clearer picture of the relationship between *Pinnularia* and *Caloneis*. In the first work of Bruder et al., published in 2008 [52], it was shown that the assumptions summarized by David Mann [56] had been confirmed. Phylogenetic analysis does not support the traditional division *Pinnularia-Caloneis*; these genera form a single monophyletic clade. In turn, *Caloneis* turned out to be non-monophyletic, its representatives (*C. lauta, C. budensis, C. amphisbaena*) forming separate lines among *Pinnularia*. For a morphological feature that corresponded with the division of phylogenetic groups, the suggestion made by Krammer and Lange-Bertalot [54] of using the degree of alveoli closure (“nearly open alveoli”, “partially closed” and “nearly closed”) was confirmed at that moment.

In the study of Souffreau et al. [39], dedicated to a time-calibrated multi-gene phylogeny of the *Pinnularia*, representatives of *Caloneis* are positioned in different clades (A and C), which confirms the heterogeneity of this genus. A more comprehensive phylogenetic analysis has been performed, and a third clade of *Pinnularia-Caloneis* has been added to the two defined by Bruder et al. [52]. The analysis of the morphological features of the obtained subclades discusses shapes of the valves and apices, external central raphe endings (linear or rounded), raphe fissures (straight or undulate), chloroplasts (H-shaped or elongated, with pyrenoids or not, etc.), and specific markings on the central area (ghost striae, fascia, wart-like bodies, etc.).

In our phylogenetic analysis, the range of *Caloneis* strains has been significantly expanded compared to previous studies and includes 17 strains. Their positions in the clades remain the same: in clade A *C. lauta* forms separate line; the suclades “caloneis1” and “caloneis2” are separated, but they have low external support (Figure 14); *C. budensis* forms lineage with *P. brebissonii* UTEX FD274 and *P*. cf. *microstauron* (B2)c in clade C; strains of *C. amphisbaena* together with two other representatives of the genus form a separate clade “caloneis 3” (Figure 14).

#### 2.2.2. Morphological Features of Some Phylogenetical Groups

Currently, it is generally quite hard to connect division into clades with any kind of morphological patterns, since species in every clade are very variable in valve size and shape. For example, clade A contains both the large-celled, morphologically close to the *viridis* group *P. valida* and the small-celled *Caloneis silicula*, *C. fontinalis*. For the most part clade B includes taxa with linear valves and capitate or subcapitate ends, but there are exceptions, like the small-celled species with an elliptic valve outline *P. microfraubergiana* sp. nov. The presence of a fascia on the central area is characteristic for all species of clade B except *P. acrosphaeria*. Clade C also includes very different forms: large, elongated elliptical *viridis*-like valves in the species from “viridiformis” and “subcommutata” subclades and small-celled *P. altiplanensis*, *Caloneis budensis,* etc.

The connection of phylogenetic groups and morphological features is more defined at the subclade level. Our further discussion is based on a study of voucher images that can be openly accessed (Table S1). For the species that do not have accessible voucher images or the images do not contain the necessary morphological features (for example, there are only live cells pictured, there are no SEM images on which the ultrastructure of the central area or the degree of closure of the alveoli could be studied, etc.), we considered taxa descriptions and features given in the relevant literature and descriptions from [39,52]. In the end, we could not find images only for 8 unidentified taxa (less than 10%) (see Table S1).

To determine the significance of a specific morphological feature as a phylogenetic signal, we compiled a comparison table for the main morphological features mentioned in previous works [39,52] and compared them with the phylogenetic groups. Unique features, i.e., those that appear in one or two clade and can be used as differentiating, are highlighted in bold (Table 8). We do not, however, speak of a 100% conclusion, since our phylogeny only includes a small part of the whole *Pinnularia*-*Caloneis* diversity, we are only making a suggestion based on an analysis of a concrete set of taxa.

So, the structure of internal alveoli aperture can be used to define subclade “caloneis1” and a monospecies subclade “acrosphaeria”, which are characterized by nearly closed alveoli. A preliminary analysis indicates that nearly closed alveoli are a rare and specific feature, the importance of which is confirmed by our phylogeny. In the future it can be used as differentiating. Generally, we can conclude that for each subclade the structure of internal alveoli aperture is a unifying feature (Table 8). Markings on the valves surface are present in representatives of five subclades, however, the ultrastructure of these markings is distinctive in each subclade.

In the subclades “divergens” and “stomatophora”, the markings are crescent-shaped or irregular hollow on the external surface of the central area. However, among the representatives of “divergens”, the presence of such markings is confirmed only for *P. baicalodivergens* sp. nov. (this study, Figure 10A,B). After studying LM images of the *P. divergens* D31_023 voucher, we can also assume the presence of these structures. Note that on the voucher images of *Caloneis* representatives from clade A we did not find such structures on the central area (Table S1). However, according to literary data, crescent-shaped hollows are shown for *Caloneis lewisii* and *C. silicula* [62,63]. In any case these crescent-shaped or irregular hollows on the external surface of the central area have been found only in representatives of clade A.

Most of the taxa from subclade “subgibba” have the so-called ghost striae (slight thinnings of the valve that correspond in size and spacing to the normal striae [64] on the internal surface of the central area. The exception are the strains of *Pinnularia* sp. 6 Tor4r, on the voucher image ghost striae are not distinct [39], p. 867, Figure 1o), and in *P. siberiosinistra* (this study, Figure 8 W,X) ghost striae are absent.

The monospecies lines “nodosa” and “acrosphaeria” have a heavily structured central area; “nodosa” has a relief-like structure on the outer surface and “acrosphaeria” has mottled, wart-like structures on the outer surface and is slightly structured on the inner surface.

A fascia is a unifying feature for the vast majority of the subclades. Subclades “caloneis1” and “borealis” are the exception. In the first one, all taxa except *Caloneis lewisii* have a fascia. In the “borealis” subclade, all taxa except *P. paradubitabilis* have a fascia present. The presence of a fascia is generally characteristic for representatives of subclades “divergens”, “stomatophora”, “subgibba”, *P. nodosa*, “grunowii”. Taxa without fascia are combined in subclades “viridiformis”, “subcommutata”, and “caloneis 2”.

Subclade “viridiformis” clearly stands out by the raphe structure, because only this clade unites taxa with a complex or semicomplex raphe. Other subclades contain species with a lateral and/or fusiform raphe. The border lineage between subclades “viridiformis” and “subcommutata” includes *P. substreptoraphe* AT 70.09 with a complex raphe and *P. acuminata* Pin876 TM with a lateral raphe, but this lineage is poorly supported (Figure 14).

Concerning the structure of the chloroplasts, most species in our analysis have two plate-like chloroplasts. The two plate-like chloroplasts are widest in girdle view, with the small parts of each edge extending to valve view. Some species with one H-shaped chloroplast are united in the subclade “grunowii”. Also, H-shaped chloroplasts are present in *P*. sp. (Wie)a, *P*. cf. *altiplanensis*, *P*. cf. *isselana* (“subcommutata” subclade), *P*. *microstauron* (“microstauron” subclade), and one species in subclade “caloneis1”—*Caloneis silicula* (according to Bruder et al. [52] and Souffreau et al. [39]). Thus, the significance of chloroplast structure for phylogenetic differentiation is not yet clear.

## 3. Conclusions

Based on our analysis presented herein, with a greater degree of taxon sampling than past studies, we can say there is better resolution of the genera *Caloneis* and *Pinnularia* and a reason to continue to recognize them as distinct. The genera *Caloneis* and *Pinnularia* are each monophyletic but not with the composition of species traditionally assigned to them. For *Caloneis*, the generitype (*C. amphisbaena*) and other species form a strongly supported monophyletic group that is distinct from *Pinnularia sensu lato*. Small species traditionally assigned to *Caloneis,* such as *C. silicula, C. lewisii, C. lauta*, etc, however, are not part of this lineage; they fall out within a broader concept of *Pinnularia*. The genus *Pinnularia s. l.* is also a monophyletic group, but it is not strongly supported. Within *Pinnularia*, the three subgroups (A, B, and C) are monophyletic, though only one in our analysis (Clade B) has strong support—however, this group does not contain the generitype (*P. viridis*). The large number of taxa described in *Pinnularia* (with over 4200 named taxa; [15]) makes it tempting to begin recognizing subgroups within *Pinnularia s.l.* as distinct genera, and assigning morphological characters to these groups supports that approach. But, further analyses and better support for the groups may be a crucial next step in the direction of creating a refined, natural classification of the Pinnlariaceae.

## 4. Materials and Methods

### 4.1. Sampling

**Figure 16:**
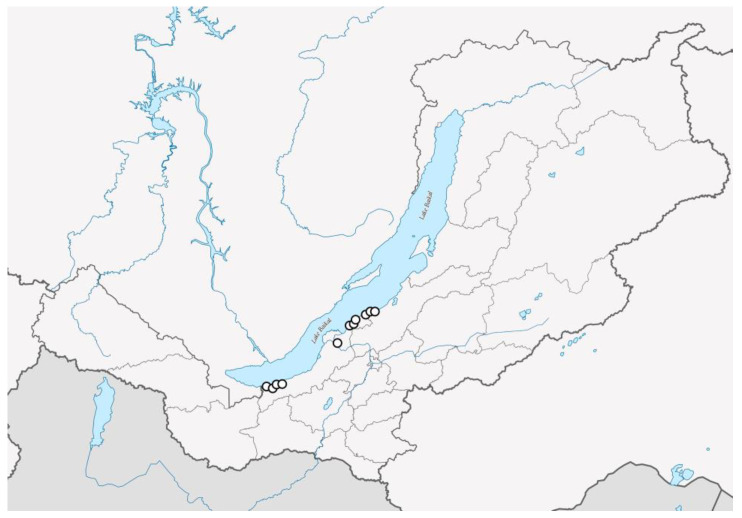
Map showing the sampling locations.

The samples used in the present report were collected from Eastern Siberia, Russia, by Maxim Kulikovskiy. The samples were collected in deltas of rivers that drain into Lake Baikal (the rivers Kapustinskaja, Selenga, Zagza, Vydrinnaja, Bolshaya Suhaya) as well as in Lake Baikal itself (see Figure 16, Table 9). Water mineralization and temperature measurements were performed using the Hanna Combo (HI 98129) multiparameter probe (Hanna Instruments, Inc., Woonsocket, RI, USA). A list of all strains examined in this study with their GenBank accession numbers and geographic location of sampling sites with measured ecological parameters is presented in Table 9.

### 4.2. Culturing

A subsample of each collection was added to WC liquid medium [65]. Monoclonal strains were established by micropipetting a single cell under an inverted microscope Axio Vert. A1 (Zeiss, Oberkochen, Germany). Non-axenic unialgal cultures were maintained in WC liquid medium at 22–25 °C in a growth chamber with a 12:12 h light:dark photoperiod. Strains were analyzed after one month of culturing.

### 4.3. Preparation of Slides and Microscope Investigation

Strains for LM and SEM investigations were processed by means of a standard procedure involving treatment with concentrated hydrogen peroxide. The material was washed with distilled water. Permanent diatom preparations were mounted in Naphrax^®^ (Brunel Microscopes Ltd., Chippenham, UK; refractive index = 1.73). Light microscopic (LM) observations were performed using the microscope AxioScope A1 (Zeiss, Germany) equipped with an oil immersion objective (×100/n.a.1.4, DIC). Ultrastructure of the valves was examined with the scanning electron microscope JSM-6510LV (Jeol, Tokyo, Japan).

### 4.4. Molecular Study

Total DNA from the studied strains was extracted using Chelex 100 Chelating Resin, molecular biology grade (Bio-Rad Laboratories, Hercules, CA, USA), according to the manufacturer’s protocol 2.2. Partial 18S rRNA (378–382 bp, including the highly variable V4 region), and partial *rbc*L plastid genes (978 bp) were amplified using primers D512for and D978rev from Zimmermann et al. [66] for 18S rDNA fragments and *rbc*L66+ and *rbc*L1255-from Alverson et al. [67] for *rbc*L fragments.

Amplifications were carried out using premade polymerase chain reaction (PCR) mastermixes (ScreenMix by Evrogen, Moscow, Russia). Amplification conditions for the 18S rRNA gene were as follows: initial denaturation for 5 min at 95 °C followed by 35 cycles of 30 s denaturation at 94 °C, 30 s annealing at 52 °C, and 50 s extension at 72 °C, with the final extension for 10 min at 72 °C. Amplification conditions for the *rbc*L gene were as follows: initial denaturation for 4 min at 94 °C followed by 40 cycles of 50 s denaturation at 94 °C, 50 s annealing at 53 °C, and 80 s extension at 72 °C, with the final extension for 7 min at 72 °C.

PCR products were visualized by horizontal electrophoresis in 1.0% agarose gel stained with SYBRTM Safe (Life Technologies, Carlsbad, CA, USA). The products were purified with a mixture of FastAP, 10× FastAP Buffer, Exonuclease I (Thermo Fisher Scientific, Waltham, MA, USA), and water. The sequencing was performed using a Genetic Analyzer 3500 instrument (Applied Biosystems, Waltham, MA, USA).

Editing and assembling of the consensus sequences were carried out by processing the direct and reverse chromatograms in Ridom TraceEdit ver. 1.1.0 (Ridom GmbH, Münster, Germany) and Mega ver. 7 software [68]. The reads were included in the alignments along with corresponding sequences of 89 diatom species downloaded from GenBank (taxa names and Accession Numbers are given in Figure 14). Five *Sellaphora* species were chosen as the outgroups.

The nucleotide sequences of the 18S rRNA and *rbc*L genes were aligned separately using the Mafft ver. 7 software (RIMD, Osaka Japan) and the E-INS-i model [69]. The final alignments were then carried out: unpaired sites were visually determined and removed from the beginning and the end of the resulting matrices. For the protein-coding sequences of the *rbc*L gene, we checked that the beginning of the aligned matrix corresponds to the first position of the codon (triplet). The resulting alignments had lengths of 450 (18S rDNA) and 1347 (*rbc*L) characters. After removal of the unpaired regions, the aligned 18S rDNA gene sequences were combined with the *rbc*L gene sequences into a single matrix Mega7.

The data set was analyzed using the Bayesian inference (BI) method implemented in Beast ver. 1.10.1 software (BEAST Developers, Auckland, New Zealand) [70] to construct a phylogeny. For the alignment partition, the most appropriate substitution model, shape parameter α, and a proportion of invariable sites (pinvar) were estimated using the Bayesian information criterion (BIC) as implemented in jModelTest ver. 2.1.10 (Vigo, Spain) [71]. This BIC-based model selection procedure selected the following models, shape parameter α and a proportion of invariable sites (pinvar): TrN + I + G, α = 0.5040 and pinvar = 0.4060 for 18S rDNA; TPM1uf + I + G, α = 0.4570, and pinvar = 0.6910 for the first codon position of the *rbc*L gene; TVMef + I + G, α = 0.2830 and pinvar = 0.7030 for the second codon position of the *rbc*L gene; TVM + I + G α = 0.8250, and pinvar = 0.1270 for the third codon position of the *rbc*L gene. We used the HKY model of nucleotide substitution instead of TrN, the GTR model instead of TPM1uf, TVMef and TVM, given that they were the best matching model available for BI. A Yule process tree prior was used as a speciation model. The analysis ran for 5 million generations with chain sampling every 1000 generations. The parameter-estimated convergence, effective sample size (ESS), and burn-in period were checked using the Tracer ver. 1.7.1 software (MCMC Trace Analysis Tool, Edinburgh, UK) [70]. The initial 25% of the trees were removed, and the rest were retained to reconstruct a final phylogeny. The phylogenetic tree and posterior probabilities of its branching were obtained based on the remaining trees, having stable estimates of the parameter models of nucleotide substitutions and likelihood. The maximum-likelihood (ML) analysis was performed using RAxML ver. 8 on XSEDE software [72]. The nonparametric bootstrap analysis with 1000 replicas was used. FigTree ver. 1.4.4 (University of Edinburgh, Edinburgh, UK) and Adobe Photoshop CC ver. 19.0 software (Adobe, San Jose, CA, USA) were used for viewing and editing the trees. Sequences from *Pinnularia* species obtained in this study were deposited to GenBank (Table 9).

## Figures and Tables

**Table 1:** Comparison of morphological features of *P. baicalgenkalii* sp. nov. and related species.

	*P. baicalgenkalii* sp. nov.	* P. genkalii *	* P. ilkaschoenfilderi *	* P. reichardtii *
Outline	elliptic with parallel sides	linear	linear	linear
Ends	broadly rounded	cuneiform rounded	cuneate and broadly rounded	linear cuneate rounded
Length, μm	92–99	75–130	75–105	72–130
Width, μm	19.5–20	17–20	13.4–17	14.7–18.8
Striae in 10 μm	6	6–7	6–7	8–9
Raphe	complex, undulate	semicomplex to broadly complex	broadly complex	broadly semicomplex
Axial area	linear, narrow	linear, narrow 1/5 width of the valve	linear 1/5–1/3 the width of the valve	linear 1/4–1/3 the width of the valve
Central area	small, asymmetrically elliptic	slight asymmetrically	large, roundish	small, asymmetrically rounded
References	This study	[38]	[38]	[38]

**Table 2:** Comparison of morphological features of *P. baicalflexuosa* sp. nov. and related species.

	*P. baicalflexuosa* sp. Nov.	*P. neglectiformis* Pin706F	* P. neglectiformis *	* P. torta *	* P. flexuosa *
**Outline**	linear with parallel sides	linear, sides parallel to slightly convex or triundulate	linear, sides parallel to slightly convex or triundulate	linear	linear, sides parallel
**Ends**	broadly rounded	rounded to cuneiform rounded	rounded to cuneiform rounded	broadly rounded	broadly rounded
**Length, μm**	109–116	101.4–109.2	80–130	127.5–189	173–270
**Width, μm**	17.5–19	18–19	16–20	20–24	32–50
**Striae in 10 μm**	7–8	7.9–8.3	8–9	6–7	4–5
**Raphe**	semicomplex, undulate	semicomplex	semicomplex	semicomplex	semicomplex to complex
**Axial area**	linear, narrow	linear 1/5–1/4 width of valve	linear 1/5–1/4 width of valve	linear, near 1/4 width of valve	linear 1/4 to 1/3 width of valve
**Central area**	small, asymmetrically elliptic	a little wider than the axial area, roundish or irregular	a little wider than the axial area, roundish or irregular	asymmetrical, rhombic-round, 1/3–1/2 width of valve	a slight widening of the axial area, asymmetrical
**References**	This study	[39]	[38]	[40,41]	[38]

**Table 4:** Comparison of morphological features of *P. pergrunowii* sp. nov. and related species.

Species, Strains	Outline	Ends	Valve Length, μm	Valve Width, μm	Striae in 10 μm	Raphe	Axial Area	Central Area	References
*P. pergrunowii* sp. nov. B162	linear, sides concave	capitate	49.5–51	8.0–8.5	10–11	straight, filiform	linear, narrow, widening towards the central area	wide transverse fascia	This study
*P. anglica*	linear, sides straight	distinctly small subcapitate	30–70	10–13	9–11	filiform, key and slot	linear, narrow	rhombic, irregular or a moderatly broad fascia	[38]
*P. mesolepta* AT_160Gel30	linear, triundulate	rostrate	43.8–46.1	8.3–8.9	11–13	lateral, key and slot raphe	narrow, slightly lanceolate	rhombic, asymmetrical fascia	[46]
*P. grunowii* Pin 889 MG	linear, triundulate	capitate	41.1–42.7	7.7–8.1	12.2–13.8	straight, filiform	linear, narrow	rhombic, fascia	[39]
*P. termitina* UTEX FD484	n/d	n/d	n/d	n/d	n/d	n/d	n/d	n/d	https://utex.org/products/utex-lb-fd-0484 (accessed on 25 September 2023)
*P. termitina*	linear, triundulate	subcapitate	31–57	4.5–5.5	11–15	straight, filiform	linear, narrow	rhombic, wide fascia	[41]
*P. rhombofasciata*	linear	capitate	56–64	9–9.4	9–10	straight, filiform	linear, narrow	rhombic, fascia	[47]
*P. ferrophila*	linear, triundulate to biundulate	broadly capitate to flatly rounded	30–62	8.8–10	9–10	moderately lateral	1/4–1/5 of the width of the valve	very large, rhombic fascia	[38]
*P. dicephala*	linear	broadly capitate	44–50	6–6.7	10–11	lateral, weakly undulate	1/3 of the width of the valve expanded towards the center	rhombic-round, wide fascia	[40]
*P. latarea*	linear, sides concave	capitate with narrow neck and broad shoulders	35–64	8–10	9–11	narrowly, lateral	form together a wide, lanceolate space with a very broad fascia		[38,48]

**Table 5:** Comparison of morphological features of *P. siberiosinistra* sp. nov. and related species.

	*P. siberiosinistra* sp. nov.	*P. sinistra*	*P*. sp. (Tor4)r
**Outline**	narrowly elliptical	linear with slightly convex, more rarely straight or weakly concave sides	linear with weakly undulate sides
**Ends**	subcapitate	indistinctly differentiated and broadly protracted	subcapitate
**Length, μm**	25–29	17–52	42.6
**Width, μm**	5	4–6.5	5.8
**Striae in 10 μm**	12–14	11–13 (14)	12.4
**Raphe**	straight, filiform	filiform, somewhat lateral in large specimens	straight, filiform
**Axial area**	narrowly lanceolate and widening towards the central area	linear, in large individuals lanceolate	lanceolate and widening towards the central area
**Central area**	presented by a wide transverse fascia	an often slightly asymmetric fascia	presented by a wide transverse fascia
**References**	This study	[38,49]	[39]

**Table 6:** Comparison of morphological features of *P. baicalodivergens* sp. nov. and related species.

	*P. baicalodivergens* sp. nov. B112	*P. baicalodivergens* sp. nov. B097	*P. divergens* var. *media*	*P. microstauron* var. *microstauron*	*P. submicrostauron*
**Outline**	linear	linear	linear	linear	linear
**Ends**	subcapitate rounded	subcapitate rounded	subcapitate rounded	broadly rostrate and wedge-shaped	rostrate rounded
**Length, μm**	46–50	43.5–50	40–70	30–78(100)	37–45
**Width, μm**	8–9	8.4–9.4	10–13	10–12.4	8–9
**Striae in 10 μm**	11–12	11–12	10–11	9–11(15)	12–13
**Raphe**	straight, filiform	straight, filiform	straight, filiform	straight, filiform	lateral
**Axial area**	linear, narrow, widening towards the central area	linear, narrow, widening towards the central area	linear, narrow (1/3), widening towards the central area	linear, narrow	linear
**Central area**	asymmetric transverse fascia	asymmetric transverse fascia	rhombic-round, fascia	asymmetric transverse fascia, often absent	rhombic-round, fascia
**References**	This study	This study	[38,48,50]	[38,40]	[40]

**Table 7:** Comparison of morphological features of *P. baicalislandica* sp. nov. and related species.

Species, Strains	Outline	Ends	Valve Length, μm	Valve Width, μm	Striae in 10 μm	Raphe	Axial Area	Central Area	References
*P. baicalislandica* sp. nov.	linear	broadly rounded	54–60	10–11	10	straight, filiform	linear, narrow	asymmetric, rhomboid	This study
*P. subcomutata*	linear-elliptic to linear-lanceolate, sides slightly convex	broadly rounded	32–83	10–13.4	9–12	lateral	linear, narrow (1/5)	roundish-rhombic or orbicular, simefascia 1–2 striae on one side are absent	[38]
*P. subcomutata* var. *nonfasciata*	linear-elliptic to linear-lanceolate, sides slightly convex	broadly rounded	32–83	10.0–13.4	9–12	lateral	linear, narrow (1/5)	roundish-rhombic or orbicular, without fascia	[38]
*P. islandica*	linear-elliptic	broadly rounded	48–84	12–14	9–10	lateral, moderately broad	up to 1/3 the width of the valve	rhombic to irregular roundish, sometimes crescent-like markings are present	[38]
*P. rupestris*	linear-elliptic	broadly rounded	40–90	9–12.4	12–13	lateral narrow to moderately broad	up to 1/4 the width of the valve	round to elongated-elliptic	[38,43]
*P. levkovii*	linear to linear-elliptic	broadly rounded	36–60	8–10	8–10	narrowly lateral	1/4 the width of the valve extended towards a central area	variably shaped, narrowly	[51]
*P. perspicua*	linear	broadly cuneate, rounded	40–65	13–15	8–11	lateral, moderately broad	linear 1/5–1/4 the width of the valve	rhomboidal with weakly developed crescent-like markings	[38,50,51]

**Table 8:** Correlation of morphological features in phylogenetic clades and subclades of *Pinnularia*-*Caloneis*.

Clade	Subclade	Internal Alveoli Aperture	Markings on the Valve Surface	Fascia	Raphe System	Chloroplasts Form
**A**	“divergens”	nearly open	none or crescent-shaped or irregular hollow markings on the external surface	+	lateral	two plate-like
	“stomatophora”	nearly open	crescent-shaped or irregular hollow markings on the external surface *	+	lateral	two plate-like
	* Caloneis lauta *	n/d	no	+	fusiform	two plate-like
	“caloneis1”	**nearly closed**	none or not confirmed on the voucher images	+/−	fusiform	two plate-like/H-shaped in *C. silicula*
	“caloneis2”	n/d	n/d	n/d	n/d	n/d
**B**	“subgibba”	partially closed	ghost striae on the internal surface of the central area **	+	lateral	two plate-like
	“nodosa”	nearly open	**heavily structured by a relief-like structure on the outer surface**	+	lateral	two plate-like
	“grunowii”	nearly open	no	+	lateral or fusiform	H-shaped
	“acrosphaeria”	**nearly closed**	**mottled, wart-like structures on outer surfaces and slightly structured on inner surfaces**	-	lateral	two plate-like
**C**	“viridiformis”	partially closed	no	-	**complex or semicomplex**	two plate-like
	*P. substreptoraphe* AT 70.09	partially closed	no	-	**complex**	two plate-like
	*P. acuminata* Pin876 TM	n/d	no	-	lateral	two plate-like
	“subcommutata”	partially closed	no	-	lateral	H-shaped
	*P.* sp. 4 (Wie)a	n/d	no	-	lateral	H-shaped
	*P. altiplanensis* Tor11b	n/d	no	+	fusiform	H-shaped
	*P. brebissonii* UTEX FD274 *P*. cf. *microstauron* (B2)c *Caloneis budensis*	partially closed	no	+	lateral/ fusiform	H-shaped/ two plate-like
	“borealis”	nearly open	no	−/+	lateral or fusiform	two plate-like
	“caloneis 3”	nearly open	no	-	fusiform	two plate-like

* Unique features highlighted in bold. ** There are exceptions.

**Table 9:** List of strains examined in this study, with their GenBank accession numbers. Geographic locality of samples and measured ecological parameters are indicated.

Strains	Slide No	Sample Locality	Collection of Date	Coordinates	t (°C)	pH	Cond. (μS cm^−1^)	Substrate	GenBank Accession Number, *rbc*L, Partial	GenBank Accession Number, SSU rDNA, Partial
*P. baicalogenkalii* B194	19179	Russia, Kapustinskaja River, flowing into Lake Baikal, near to the cape Tolstoj, sample no 34	17 July 2011	52°38.484′ N 107°23.218′ E	9.5	6.9	61	benthos	KM350002	KM350092
*P. baicaloflexuosa* B054–3	18959	Russia, Selenga River, near the Brjansk Village, sample no 40	19 July 2011	52°03.376′ N 106°52.672′ E	25	7.9	202	benthos	KM349984	KM350068
*P. microfrauenbergiana* B025	18931	Russia, Vydrinnaja River, sample no 51.1	20 July 2011	51°29.383′ N 104°50.986′ E	7	n/d	14	periphyton	KM349979	KM350062
*P. pergrunowii* B162–3	18989	Russia, Bolshaya Suhaya River near to the Zarech’e Settlement, sample no 28.2	17 July 2011	52°33.418′ N 107°08.564′ E	15.4	6.1	63	benthos	KM349996	KM350084
*P. siberiosinistra* B024–1	18930	Russia, Vydrinnaja River, sample no 51.1	20 July 2011	51°29.383′ N 104°50.986′ E	7	n/d	14	periphyton	KM 349978	KM 350061
*P. baicalodivergens* B112	19170	Russia, unnamed Bay in 8 km from Jenhaluk, sample no 4.5	14 July 2011	52°27.042′ N 106°53.215′ E	25	7.5	295	benthos	KM349992	KM350078
*P. baicalodivergens* B097	19248								KM349990	KM350075
*P. baicalislandica* B238	18997	Russia, Zagza River, sample no 11.2	15 July 2011	52°31.656′ N 107°05.114′ E	14	8.5	40	periphyton	KM350009	KM350101
P. *sp.*, B027–1	19223	Russia, Vydrinnaja River, sample no 51.1	20 July 2011	51°29.383′ N 104°50.986′ E	7	n/d	14	periphyton	KM349981	KM350064
*P. parvulissima* B028	19222	Russia, Vydrinnaja River, sample no 51.1	20 July 2011	51°29.383′ N 104°50.986′ E	7	n/d	14	periphyton	KM349982	KM350065
*P*. sp., B026–1	18941								KM349980	KM350063
*P. subgibba* var. *sublinearis* B296–1	18922	Russia, Zagza River, sample no 11.2	15 July 2011	52°31.656′ N 107°05.114′ E	14	8.5	40	periphyton	KM350013	KM350110
*C. fontinalis strain* B182	19015	Russia, Kapustinskaja River, flowing into Lake Baikal, near to the cape Tolstoj, sample no 34	17 July 2011	52°38.484′ N 107°23.218′ E	9.5	6.9	61	benthos	KM349999	KM350087

## Data Availability

Not applicable.

## Supplementary Materials

The following supporting information can be downloaded at: https://www.mdpi.com/article/10.3390/plants12203552/s1, Table S1: Strain list and reference to voucher or publication nucleotide sequence used in phylogenetic analysis. References [73,74,75,76,77,78,79,80,81] are cited in Supplementary Materials.
