# Supplementary material for: The Genus Pinnularia Ehrenberg (Bacillariophyta) from the Transbaikal Area (Russia, Siberia): Description of Seven New Species on the Basis of Morphology and Molecular Data with Discussion of the Phylogenetic Position of Caloneis
Source: Plants (Basel). 2023 Oct 12;12(20):3552. doi: 10.3390/plants12203552 (PMC10610464; doi:10.3390/plants12203552)
Supplement: Supplementary file 1 [file plants-12-03552-s001.zip › plants-2611526-supplementary.pdf]

Supplementary Table S1. Strains list and reference to voucher or publication nucleotide sequence used in phylogenetic analysis. The "Reference to voucher" column contains direct links to images in databases (Algaterra, UTEX) or publications. The "Reference to image if there is no voucher" column contains reference to publications with images of taxa that we used if vouchers were not available. The "No image or voucher" column indicates references to publications related to nucleotide sequences (but images are not available). * The reference is given after the table.

**№**	**Taxon**	**Strain**	**Reference to a voucher**	**Reference to an image if there is no voucher**	**No image or voucher (reference to publication of the nucleotide sequence)**
1	*P.* sp. 1 (divergens-group)	(Tor1)b	Souffreau et al. 2011 [39], p.867, fig. 1f		
2	P. sp. 7 (divergens-group)	(Tor7)c	Souffreau et al. 2011 [39], p.867, fig. 1e		
3	P. sp.	B027-1	This study, fig. 15 A-C		
4	*P. baikalodivergens* Kulikovskiy, Glushchenko, Kezlya & Maltsev sp. nov.	B112	This study, fig. 9, 10		
5	*P. baikalodivergens* Kulikovskiy, Glushchenko, Kezlya & Maltsev sp. nov.	B097	This study, fig. 11		
7	P. *divergens* W.Smith	D31_023		Krammer 2000 [38]	
8	*P. valida* Hustedt	VN305	-	Kulikovskiy et al. (unpublished)	
9	*P. ministomatophora* Kezlya, Maltsev, Krivova et Kulikovskiy	VP563	Kezlya et al. 2022, fig. 12		
10	*P. stomatophora* (Grunow) Cleve	D11-014	-	Krammer 2000 [38]	
11	*Caloneis lauta* Carter and Bailey-Watts	AT160 Gel04		Carter, J., Bailey-Watts 1981	
12	*C. fontinalis* (Grunow) A.Cleve	D05-043		Van de Vijver et al. 2020 https://www.researchgate.net/publication/341901607_Observations_on_and_typification_of_Navicula_fontinalis_Grunow_Naviculaceae_Bacillariophyta/figures?lo=1	
13	*C. fontinalis* (Grunow) A.Cleve	D05-042	-		
14	*C. fontinalis* (Grunow) A.Cleve	D05-040	-		

15	*C. fontinalis* (Grunow) A.Cleve	B182	This study, fig. 15 G-I		
16	*C.* sp	NE S01 D04-013	-	-	unpublished
17	*C. silicula* (Ehrenberg) Cleve	D06-074		Krammer, K., Lange-Bertalot 1986	
18	*C. silicula* (Ehrenberg) Cleve	Cal890 TM	Souffreau et al. 2011 [39], p.868, fig. 2a,b		
19	*C. lewisii* R.M.Patrick	UTEX FD54	https://utex.org/products/utex-lb-fd-0054	Spaulding et al. 2021[62]	
20	*C.* sp	KSA2015	-		Sabir et al. 2018*
21	*C.* cf. *linearis* (Cleve) Boyer	21IV14 3A	-		Lobban et al. 2021*
22	*Pinnularia parvulissima* Krammer	B028	This study, fig. 15 D-F		
23	*P.* cf. *gibba*		-	Krammer 2000 [38]	Evans et al. 2008*
24	*P. microstauron* (Ehrenberg) Cleve	AT 113Gel11		Krammer 2000 [38]	
25	*P.* sp.	B026-1	This study, fig. 15 J-M		
26	*P. subgibba* var. *sublinearis* Krammer	26JZ01-4	-	Krammer 2000 [38], plate 65, p.389, fig.14	
27	*P. microstauron* (Ehrenberg) Cleve	AT69.06		Krammer 2000 [38]	
28	*P.* cf. *subgibba* var. *sublinearis* Krammer	B296-1	This study, fig. 15 N-Q		
29	*P. parvulissima* Krammer	Pin877	Souffreau et al. 2011 [38], p.867, fig. 1l.		
30	*P.* sp. 3	(Tor8)b	Souffreau et al. 2011 [38], p.867, fig. 1p.		
31	*P.* sp. 2	(Tor7)f	Souffreau et al. 2011 [38], p.867, fig. 1m.		
32	*P. kattiensis*	VN314	-	Kulikovskiy et al. (unpublished)	
33	*P. kattiensis*	VN354	-	Kulikovskiy et al. (unpublished)	
34	*P. subcapitata* var. *elongata* Krammer	(Wie)c	Souffreau et al. 2011 [38], p.867, fig. 1n.		
35	*P.* sp. 6	(Tor4)r	Souffreau et al. 2011 [38], p.867, fig. 1o.		
36	*P.* sp. 8	PinnC7	-		Souffreau et al. 2011[38]

37	*P. siberiosinistra* Kulikovskiy, Glushchenko, Kezlya & Maltsev sp. nov.	B024-1	This study, fig. 8		
38	*P. vietnamogibba* Kezlya, Maltsev, Krivova et Kulikovskiy	VP290	Kezlya et al. 2022 [53], fig 5 J–O		
39	*P. vietnamogibba* Kezlya, Maltsev, Krivova et Kulikovskiy	VP294	Kezlya et al. 2022 [53], fig 5 A–E,6		
40	*P. minigibba* Kezlya, Maltsev, Krivova et Kulikovskiy	VP284	Kezlya et al. 2022 [53], fig 3,4		
41	*P. shivae* Cejudo-Figueiras, S.Blanco & Álvarez-Blanco	VN399	-	Blanko et al. 2012, fig. 9-13	
42	*P. microgibba* Kezlya, Maltsev, Krivova et Kulikovskiy	VP289	Kezlya et al. 2022 [53], fig 7 A– J, 8		
43	*P. microgibba* Kezlya, Maltsev, Krivova et Kulikovskiy	VP292	Kezlya et al. 2022 [53], fig 7 (L–S)		
44	*P. nodosa* (Ehrenberg)W.Smith	Pin885 TM	Souffreau et al. 2011 [38], p.867, fig. 1k, Krammer 2000 [37], plate 24, fig. 1-14, plate 25, ffig. 1-14,		
45	*P. pergrunowii* Kulikovskiy, Glushchenko, Kezlya & Maltsev sp. nov.	B162-3	This study, fig. 7		
46	*P. grunowii* Krammer	Pin889 MG	-	Krammer 2000 [37] plate 77, fig. 7-14, plate 81, fig. 10-15, plate 82 fig. 7,8	Souffreau et al. 2011 [38]
47	*P. mesolepta* (Ehrenberg) W. Smith	AT 160Gel30		Krammer 2000 [38]	
48	*P. termitina* (Ehrenberg) R.M.Patrick	UTEX FD484			https://utex.org/products/utex-lb-fd-0484
49	P. *anglica* Krammer	AT 100Gel01		Krammer 2000 [38]	
50	*P*. sp. 5	(Tor4)i	Souffreau et al. 2011 [38], p.867, fig. 1g		
51	*P*. cf. *marchica* lka Schönfelder	Ecrins4a	Souffreau et al. 2011 [38], p.867, fig. 1i		
52	*P. obscura* Krasske	AT 70Gel12b		Krammer 2000 [38]	
53	*P. microfrauenbergiana* Kulikovskiy, Glushchenko, Kezlya & Maltsev sp. nov.	B025	This study, fig. 6		

54	*Pinnularia insolita* Kezlya, Maltsev, Krivova et Kulikovskiy s	VP280	Kezlya et al. 2022 [51], fig 9,10		
55	*P. subanglica*	Pin650	Souffreau et al. 2011 [39], p.867, fig. 1h		
56	*P. acrosphaeria* W. Smith	D25_019	-	Krammer 2000 [38], plate 19, fig. 1-6, plate 20. fig.5-7, plate 21, fig. 1-7	
57	*P. acrosphaeria* W. Smith	26JZ01-3	-	Krammer 2000 [38], plate 19, fig. 1-6, plate 20. fig.5-7, plate 21, fig. 1-7	
58	*P. neomajor* Krammer	Corsea 2	-	Krammer 2000 [38]	Souffreau et al. 2011 [39]
59	*P. neomajor* Krammer	AmPi02	-	Krammer 2000 [38]	
60	*P. neomajor* Krammer	B029		Krammer 2000 [38]	
61	*P. viridiformis* Krammer	Pin870 MG	-	Krammer 2000 [38]	Souffreau et al. 2011 [39]
62	*P. viridis* Krammer	D11_043	-	Krammer 2000 [38]	
63	*P. viridiformis* Krammer	(Enc2)a	Souffreau et al. 2011 [39], p.867, fig. 1r	Krammer 2000 [38]	
64	*P. neomajor* Krammer	(Tor1)a	-	Krammer 2000 [38]	Souffreau et al. 2011[39]
65	*P. viridiformis* group Krammer	B125	-	Krammer 2000 [38]	
66	*P. viridiformis* Krammer	AmPi01	-	Krammer 2000 [38]	
67	*P*.sp. 9 (subcommutata-group)	Pin 883 TM	-		Souffreau et al. 2011 [39]
68	*P. neglectiformis* Krammer	Pin 706 F	-	Krammer 2000 [38]	Souffreau et al. 2011 [39]
69	*P*.sp. 11 (subcommutata-group)	Pin649 K	-		Souffreau et al. 2011[39]
70	*P. viridis* Krammer	AT 161.02	-	Krammer 2000 [38]	
71	*P. baicalgenkalii* Kulikovskiy, Glushchenko, Kezlya & Maltsev sp. nov.	B194	This study, fig. 1,2		
72	*P. baicalflexuosa* Kulikovskiy, Glushchenko, Kezlya & Maltsev sp. nov.	B054-3	This study, fig. 3,4,5		

73	*P. substreptoraphe* Krammer	AT 70.09	http://www.algaterra.net/ATDB/Pictures/PicturePage.cfm?NameId=18315&RefId=900		
74	*P. acuminata* W. Smith	Pin876 TM	Souffreau et al. 2011[38], p.867, fig. 1s		
75	*P.* cf. *isselana* Krammer	Cal 878 TM	-	Krammer 2000 [38]	Souffreau et al. 2011[39]
76	*P. subcommutata* var. *nonfasciata* Krammer	Corsea10	Souffreau et al. 2011 [38], p.867, fig. 1t		
77	P. sp. 10	Pin 873 TM	-		Souffreau et al. 20113[39]
78	*P. baicalislandica* Kulikovskiy, Glushchenko, Kezlya & Maltsev sp. nov.	B238	This study, fig. 12, 13		
79	*P.* sp. 4	(Wie)a	Souffreau et al. 2011 [39], p.867, fig. 1t		
80	*P.* cf. *altiplanensis* Lange-Bertalot	(Tor11)b	Souffreau et al. 2011 [39], p.867, fig. 1x		
81	*P. brebissonii* (Kützing) Rabenhorst	UTEX LB FD274	https://utex.org/products/utex-lb-fd-0274		
82	*P.* cf. *microstauron* (Ehrenberg) Cleve (''southern microstauron'')	(B2)c	Souffreau et al. 2011 [39], p.867, fig. 1v		
83	*Caloneis budensis* Grunow) Krammer	AT 220.06	Bruder et al. 2008 [50] p. 22, fig. 11 g-h, o-p.		
84	*Pinnularia borealis* complex	REU16_05_15	-	Krammer 2000 [38]	Pinseel et al. 2020*
85	*P. paradubitabilis* Kezlya, Maltsev, Krivova et Kulikovskiy	VP236	Kezlya et al. 2022 [51] fig.13,14		
86	*P. borealis* Ehrenberg var. *subislandica* Krammer	(Tor3)a	Souffreau et al. 2013 [39], fig. 1E		
87	*P. borealis* Ehrenberg	Alka 1	Souffreau et al. 2011[39], p.867, fig. 1w 88		
88	*P. borealis* Ehrenberg cf. var. *subislandica* Krammer	(Tor12)d	Souffreau et al. 2013*, fig. 1A		
89	*Caloneis amphisbaena* (Bory) Cleve	Navi1	Zimmerman et al. 2014, p. 13, fig.3.19		

90	*C. amphisbaena* (Bory) Cleve	27JZ01-14	-	Zimmerman et al. 2014*, p. 13, fig.3.19	Unpublished
91	*C. amphisbaena* (Bory) Cleve	ElCal01a	-	Zimmerman et al. 2014*, p. 13, fig.3.19	Zimmerman et al. 2014*
92	*C.* cf. *westii* (W.Smith) Hendey	SZCZCH1002	Witkowski et al. 2016*, p. 175, fig. 3e		
93	*C.* sp.	21IV14 6A	-		Witkowski et al. 2016*

References

1. Carter, J.; Bailey-Watts, A. A taxonomic study of diatoms from standing freshwaters in Shetland. *Nova Hedwig.* **1981**, *33*, 513–629
2. Evans, K.; Wortley, A.; Simpson, G.; Chepurnov, V.; Mann, D. A molecular systematic approach to explore diversity within the Sellaphora pupula species complex (Bacillariophyta) 1. *J. Phycol*. **2008**, *44*, 215–231. https://doi.org/10.1111/j.1529-8817.2007.00454.x.
3. Lobban, C.; Majewska, R.; Ashworth, M.; Bizsel, N.; Bosak, S.; Kooistra, W.H.; Lam, D.W.; Navarro, J.N.; Pennesi, C.; Sato, S.; Van de Vijver, B. Diatom genus Hyalosira (Rhabdonematales emend.) and resolution of its polyphyly in Grammatophoraceae and Rhabdonemataceae with a new genus, Placosira, and five new Hyalosira species. *Protist* **2021**, *172*, 125816. https://doi.org/10.1016/j.protis.2021.125816.
4. Krammer, K.; Lange-Bertalot, H. Bacillariophyceae. 1. Teil: Naviculaceae. In *Süsswasserflora von Mitteleuropa*; Ettl, H., Gerloff, J., Heynig, H., Mollenhauer, D., Eds.; Gustav Fisher Verlag: Jena, Germany, 1986; 876p.
5. Pinseel, E.; Janssens, S.B.; Verleyen, E.; Vanormelingen, P.; Kohler, T.J.; Biersma, E.M.; Sabbe, K.; Van de Vijver, B.; Vyverman, W. Global radiation in a rare biosphere soil diatom. *Nat. Commun.* **2020**, *11*, 2382. https://doi.org/10.1038/s41467-020-16181-0
6. Sabir, J.; Theriot, E.; Manning, S.; Al-Malki, A.; Khiyami, M.; Al-Ghamdi, A. et alPhylogenetic analysis and a review of the history of the accidental phytoplankter, *Phaeodactylum tricornutum* Bohlin (Bacillariophyta). *PLoS ONE* **2018**, *13*, e0196744. https://doi.org/10.1371/journal.pone.0196744.
7. Souffreau, C.; Vanormelingen, P.; Van de Vijver, B.; Isheva, T.; Verleyen, E.; Sabbe, K.; Vyverman, W. Molecular evidence for distinct Antarctic lineages in the cosmopolitan terrestrial diatoms *Pinnularia borealis* and *Hantzschia amphioxys*. *Protist* **2013**, *164*, 101–115. http://doi.org/10.1016/j.protis.2012.04.001.
8. Witkowski, A.; Li, C.; Zgłobicka, I.; Yu, S.X.; Ashworth, M.; Dąbek, P.; Qin, S.; Tang, C.; Krzywda, M.; Ruppel, M.; et al. Multigene assessment of biodiversity of diatom (Bacillariophyceae) assemblages from the littoral zone of the Bohai and Yellow Seas in Yantai Region of Northeast China with some remarks on ubiquitous taxa. *J. Coast. Res.* **2016**, *74*, 166–195.
9. Zimmermann, J.; Abarca, N.; Enk, N.; Skibbe, O.; Kusber, W.; Jahn, R. Taxonomic Reference Libraries for Environmental Barcoding: A Best Practice Example from Diatom Research. *PLoS ONE* **2014**, *9*, e108793. https://doi.org/10.1371/journal.pone.0108793.
